# Latent Tuberculosis Infection Testing Strategies for HIV-Positive Individuals in Hong Kong

**DOI:** 10.1001/jamanetworkopen.2019.10960

**Published:** 2019-09-06

**Authors:** Ngai Sze Wong, Kenny Chi Wai Chan, Bonnie Chun Kwan Wong, Chi Chiu Leung, Wai Kit Chan, Ada Wai Chi Lin, Grace Chung Yan Lui, Kate M. Mitchell, Shui Shan Lee

**Affiliations:** 1 Stanley Ho Centre for Emerging Infectious Diseases, The Chinese University of Hong Kong, Shatin, Hong Kong, China; 2 Special Preventive Programme, Centre for Health Protection, Department of Health, Hong Kong Special Administrative Region Government, Hong Kong, China; 3 Hong Kong Tuberculosis, Chest, and Heart Diseases Association, Hong Kong, China; 4 Department of Medicine and Therapeutics, The Chinese University of Hong Kong, Shatin, Hong Kong, China; 5 Department of Infectious Disease Epidemiology, Imperial College London, London, United Kingdom

## Abstract

**Importance:**

With immune recovery following early initiation of antiretroviral therapy (ART), the risk of tuberculosis (TB) reactivation among individuals with HIV could be reduced. The current strategy of annual latent TB infection (LTBI) testing should be revisited to increase cost-effectiveness and reduce the intensity of testing for individuals.

**Objective:**

To analyze the cost-effectiveness of LTBI testing strategies for individuals in Hong Kong with HIV who had negative LTBI test results at baseline.

**Design, Setting, and Participants:**

This decision analytical model study using a cost-effectiveness analysis included 3130 individuals with HIV in Hong Kong, China, which has an intermediate TB burden and a low incidence of HIV-TB coinfection. A system dynamics model of individuals with HIV attending a major HIV specialist clinic in Hong Kong was developed and parameterized by longitudinal clinical and LTBI testing records of patients during a 15-year period. The study population was stratified by age group, CD4 lymphocyte level, ART status, and right of abode. Alternative strategies for LTBI testing after a baseline test were compared with annual testing under different coverages of ART, LTBI testing, and LTBI treatment scenarios in the model. An annual discounting rate of 3.5% was used in cost-effectiveness analysis.

**Main Outcomes and Measures:**

Proportion of new TB cases averted above base case scenario, discounted quality-adjusted life-years gained (QALYG), incremental cost, and incremental cost-effectiveness ratios in 2017 to 2023.

**Results:**

A total of 3130 patients with HIV (2740 [87.5%] male and 2800 [89.5%] younger than 50 years at HIV diagnosis) with 16 630 person-years of follow-up data from 2002 to 2017 were analyzed. Of these, 94 patients (0.67 [95% CI, 0.51-0.91] per 100 person-years) developed TB. Model estimates of cumulative number of TB cases would reach 146 by 2023, with the annual number of new TB diagnoses ranging from 6 to 8. For patients who had negative LTBI test results at baseline, subsequent LTBI testing strategies were ranked by ascending effectiveness as follows: (1) no testing, (2) test by risk factors, (3) biennial testing for all, (4) up to 3 tests for all, and (5) annual testing for all. Applying a willingness-to-pay threshold of $50 000 per QALYG, none of the subsequent testing strategies were cost-effective. Test by risk factors and up to 3 tests for all were cost-effective only if the willingness-to-pay threshold was increased to $100 000 per QALYG and $200 000 per QALYG, respectively. More new TB cases would be averted by expanding LTBI testing and/or treatment coverage.

**Conclusions and Relevance:**

Changing the current testing strategy to less intense testing strategies is likely to be cost-effective in the presence of an increased coverage of baseline LTBI testing and/or treatment.

## Key Points

**Question:**

In a setting with low HIV-tuberculosis incidence, is repeated testing for latent tuberculosis infection (LTBI) cost-effective for managing individuals with HIV who have negative LTBI test results at baseline?

**Findings:**

In this decision analytical model using a cost-effectiveness analysis, based on 3130 HIV-positive individuals in Hong Kong, China, a strategy of baseline LTBI testing followed by up to 3 subsequent annual tests could avert a similar proportion of new tuberculosis infections while incurring a lower cost compared with annually repeated testing. The strategy did not meet the willingness-to-pay threshold but would likely be cost-effective if the threshold were raised.

**Meaning:**

The findings suggest that less intense subsequent LTBI testing strategies may be effective and are likely cost-effective.

## Teaser

This decision analytical model analyzes the cost-effectiveness of latent tuberculosis infection testing for individuals in Hong Kong with HIV who had negative latent tuberculosis infection test results at baseline.

## Introduction

The risk of tuberculosis (TB) disease development among HIV-positive patients has been reported to be 26-fold higher than among HIV-negative individuals.^1^ To reduce TB reactivation risk, latent TB infection (LTBI) testing and treatment is the recommended HIV management strategy in areas with a low TB burden.^2^ Besides baseline LTBI screening, annually repeated testing^3^ can identify new TB infections or LTBI cases that previously had false-negative test results because of low immunity.^4^ With immune recovery in HIV-positive patients following antiretroviral therapy (ART),^5^ the risk of TB disease decreases. A study in Canada observed a decrease in TB incidence in HIV-positive individuals following increasing ART coverage from 2003 to 2012.^6^ In the United Kingdom, LTBI screening of patients with HIV based on risk factors, such as low CD4 lymphocyte level, country of origin, and duration of ART, is recommended.^7^ A study in Belgium concluded with similar doubts about the need for regular LTBI testing in HIV-positive individuals in places with a low TB burden.^8^ In the United States, in accordance with guidelines published in 2016,^9^ HIV-positive individuals belong to a high-risk category that warrants LTBI screening without subcategorization by risk.

Different strategies of LTBI testing following negative baseline test results have not been fully evaluated. The associations between LTBI screening, baseline LTBI prevalence, impact of immunological recovery on rate of TB reactivation and LTBI detection, and other factors are complex. Mathematical modeling is a useful approach to analyze the association of LTBI testing strategies with the incidence of TB. To our knowledge, the number of modeling studies addressing this issue to date has been very limited. An LTBI cost-effectiveness analysis in the United States^10^ compared LTBI testing and treatment strategies for nonlocal residents with and without medical comorbidities. The study concluded that LTBI screening for HIV-positive individuals not born in the United States was cost-effective.^10^ In the United Kingdom, universal LTBI testing was considered expensive, and there was a need for an alternative.^11^

We hypothesized that annually repeated LTBI testing for HIV-positive individuals with a negative baseline LTBI test result is not cost-effective in Hong Kong, China, a city with an intermediate TB burden and a TB incidence rate among the HIV-positive population of 0.13 per 100 person-years (PYs).^12^ At a time when immediate ART initiation is being implemented locally and internationally, this study aimed to compare the cost-effectiveness of different LTBI testing strategies for HIV-positive individuals after baseline screening.

## Methods

### Setting

The study population is a cohort of HIV-positive individuals attending the Integrated Treatment Centre, the major HIV specialist clinic in Hong Kong. After diagnosis of HIV, LTBI testing (mainly by tuberculin sensitivity testing [TST], with a small proportion by interferon-gamma release assay) is performed at baseline, then annually until diagnosis with TB or a positive LTBI test.^3,12^ Patients with positive LTBI test results (ie, >5mm induration within 48-72 hours with TST or positive interferon-gamma release assay results) are treated with isoniazid for 9 months.^3^

### Data Source

Empirical clinical data for patients diagnosed with HIV between January 2002 and June 2017 were accessed retrospectively. Data fields included baseline demographics (ie, age at HIV diagnosis, sex, race/ethnicity, and locality), baseline HIV status (ie, baseline CD4 lymphocyte, CD8 lymphocyte, and viral load levels), clinical status (ie, diabetes diagnosis and body mass index [calculated as weight in kilograms divided by height in meters squared]), first AIDS-defining illness, ART initiation date, TB diagnosis and treatment records, LTBI testing dates and results, LTBI treatment, and longitudinal CD4 lymphocyte, CD8 lymphocyte, and viral load measurements at follow-ups. Patients with HIV with a history of TB, with concurrent active TB disease, and younger than 18 years at HIV diagnosis were excluded.

Data access approval from the Department of Health, Hong Kong Special Administrative Region Government in compliance with the Personal Data (Privacy) Ordinance and ethical approval of the Joint Chinese University of Hong Kong–New Territories East Cluster Clinical Research Ethics Committee (CREC) were obtained. Individual consent was waived because the data were deidentified and had been collected retrospectively. The analysis was conducted according to Consolidated Health Economic Evaluation Reporting Standards (CHEERS) reporting guideline.

### Statistical and Cost-effectiveness Analysis

We calculated TB incidence rates (cases per PYs) with 95% CIs assuming Poisson distribution. Descriptive statistics were displayed to summarize the characteristics of patients. The test for calculating incidence was 2-sided, with statistical significance set at .05.

#### Model Structure and Assumptions

System dynamics modeling with elements of stocks, flows, feedback loops, and time delays was applied. In the model, a stock represents accumulations, and a flow is the rate of change in a stock. The methodology was used for understanding nonlinear behavior of complex systems over time, an approach that had been adopted in studying the associations of tobacco exposure with TB^13^ and optimizing an HIV testing program in Canada.^14^

We developed the model in R version 3.5.1 (The R Foundation) to simulate the flow of HIV-positive individuals with LTBI progressing to TB reactivation and HIV-positive individuals without LTBI. In the base case model, the annual number of LTBI cases was the function of new HIV diagnoses and prevalence of LTBI for HIV-positive individuals by right of abode. After HIV diagnosis, LTBI might continue to be undetected before the first LTBI test (eAppendix and eFigure 1 in the Supplement). A proportion of LTBI cases that tested positive would receive LTBI treatment, which could reduce the risk of TB reactivation.^15^ For patients who had negative LTBI test results at the first test, subsequent annual LTBI tests were performed. Cases that had positive LTBI results in subsequent tests would be offered LTBI treatment. Model parameters and equations are shown in the eAppendix, eTable 1,^2,3,16,17,18,19,20^ eTable 2, and eFigures 2-6 in the Supplement.

From our statistical analyses on the same data set, CD4 lymphocyte level, ART status, right of abode, and LTBI testing and treatment were important risk factors for TB development.^21^ As the study population was relatively young, age by itself was not a significant risk factor for TB incidence. Tuberculosis reactivation rates by right of abode, ART status, CD4 lymphocyte level, and history of LTBI treatment were determined for model parameterization (eTable 3 in the Supplement). However, recovery of CD4 lymphocyte level after ART initiation varied among age groups.^22^ We therefore divided the study population into 24 states based on age group, CD4 lymphocyte level, ART status, and residence locality (by right of abode) and stored these states in stocks, some of which change over time at different rates of flow (eFigure 1 in the Supplement). We assumed all patients would be receiving ART after TB diagnosis. However, we assumed no change of residence locality. We assumed 1.6% of TB cases were cases of multidrug-resistant TB.^17^

#### Model Outcomes

Modeling outcomes included estimated annual number of TB cases, proportion of new TB cases averted above the base case scenario, discounted incremental cost (in 2017 USD), discounted quality-adjusted life-years gained (QALYG), and incremental cost-effectiveness ratios (ICERs) in 2017 to 2023. The annual number of TB cases diagnosed in the clinic from 2002 to 2015 was used for model validation.

#### Cost and Utility Assumptions

Cost for TST was estimated for LTBI testing. For patients with positive LTBI test results, the costs of TB testing, LTBI treatment, and LTBI monitoring were included in the analysis (eTable 2 in the Supplement).^3^ For active TB cases, the costs of TB treatment and monitoring as well as multidrug resistant TB treatment and monitoring were estimated.^18^ The costs for routine HIV care were not included, as every patient was in care. The utility of HIV-positive patients without TB was assumed as 1. Loss of utility was 0.17 for newly developed, active TB cases with CD4 lymphocyte level of 200/μL or higher, 0.298 for newly developed, active TB cases with CD4 lymphocyte levels less than 200/μL, and 0.32 for multidrug-resistant TB regardless of CD4 lymphocyte level in the previous year.^19,20^ The annual inflation rate was assumed to be 2.3%,^23^ and the annual discount rate was assumed to be 3.5% for cost and quality-adjusted life-years in the analysis. Testing strategies were ranked by effectiveness, with comparisons made between each strategy and the next best alternative strategy for an ICER calculation after the exclusion of the strongly dominated and extendedly dominated strategy.^24,25^ A strategy was considered strongly dominated when it cost more but had negative QALYG compared with an alternative strategy. If the strategy had a higher ICER than a more effective strategy, it was considered extendedly dominated. A strategy was likely cost-effective if the ICER was below the willingness-to-pay (WTP) threshold, $50 000 per QALYG.^26^

#### Scenario Development and Sensitivity Analyses

We developed scenarios for comparing different subsequent LTBI testing strategies (ie, strategy A, no testing; strategy B, risk-based testing; strategy C, biennial testing; strategy D, up to 3 annual tests; and strategy E, annual testing) for patients who had negative LTBI test results at baseline. These strategies were compared under different scenarios of coverage rates (ie, scenario 1, baseline coverage levels; scenario 2, 100% coverage of ART, LTBI testing and treatment, and TB treatment; scenario 3, 100% coverage of ART, LTBI treatment, and TB treatment with baseline values for LTBI testing; scenario 4, 100% coverage of LTBI and TB treatment with baseline values for ART and LTBI testing; and scenario 5, 100% coverage of LTBI testing, LTBI treatment, and TB treatment with baseline values for ART) (Table 1).

**Table 1.** Testing Strategies for LTBI Crossed With Coverage Scenarios

Coverage Scenario^a^	New TB Cases Averted, %				
	A, No Subsequent Testing	B, Subsequent Annual Testing by Risk	C, Biennial Testing for All Until Positive Result	D, Up to 3 Subsequent Tests	E, Subsequent Annual Testing for All Until Positive Result
1, Baseline value, ie, annual coverage of 80% for ART, 51.52% for baseline LTBI testing, 42.65% for follow-up LTBI testing, and 64.86% for LTBI treatment	−4	−2	−1	−0.003	NA
2, 100% Coverage of ART, LTBI testing, and LTBI treatment	34	37	39	41	41
3, 100% Coverage of ART and LTBI treatment with baseline values for LTBI testing	9	10	11	13	13
4, 100% Coverage of LTBI treatment with baseline values for coverage of ART and LTBI testing	5	7	8	9	9
5, 100% Coverage of LTBI testing and treatment with baseline value for coverage of ART	31	36	37	39	39

Abbreviations: ART, antiretroviral therapy; LTBI, latent tuberculosis infection; NA, not applicable; TB, tuberculosis.

^a^ Scenarios 2 to 5 assume 100% TB treatment coverage.

To examine the impact of new TB infections after HIV diagnosis, we developed an expanded model by including new TB infections after HIV diagnosis and baseline LTBI testing (eFigure 7 and eFigure 8 in the Supplement). Compared with the status quo (scenario E1), the impact of reduced numbers of newly diagnosed HIV patients, change of local LTBI prevalence, and the reduction of TB reactivation risk by LTBI treatment were examined in sensitivity analyses. We performed 2-way sensitivity analyses to assess the association of LTBI testing and treatment coverage with cost-effectiveness in scenario E1 (status quo), B2 (100% coverage of ART, LTBI testing and treatment, and TB treatment with risk-based testing), and D2 (100% coverage of ART, LTBI testing and treatment, and TB treatment with up to 3 subsequent tests). Probabilistic sensitivity analysis was performed with 10 000 simulations for each LTBI testing strategy to explore the uncertainty of key parameters.

## Results

### Characteristics of Diagnosed HIV Patients

Between 2002 and 2017, 3130 HIV-positive patients with a total of 16 630 PYs of follow-up attended the Integrated Treatment Centre, of whom 2740 (87.5%) were male, 2796 of 3123 (89.5%; data for 7 patients missing) local residents, 2450 (78.3%) ethnic Chinese, and 2800 (89.5%) younger than 50 years at diagnosis of HIV. Among all patients without or before TB diagnosis, 2697 (86.2%) had initiated ART, 99 (3.2%) had been diagnosed with diabetes, and 373 (11.9%) had been diagnosed with AIDS-defining illness. After HIV diagnosis, 2412 (77.1%) had been tested for LTBI, of whom 631 (26.2%) had positive test results. Of patients with positive LTBI tests, 504 (79.9%) initiated treatment. A total of 94 patients were diagnosed with TB during the 15-year observation period.

In the cohort of patients diagnosed with HIV between 2002 and 2013 (n = 2079), the overall TB incidence was 0.67 (95% CI, 0.54-0.81) per 100 PYs. The incidence was 1.26 (95% CI, 0.95-1.63) per 100 PYs from HIV diagnosis to ART initiation, decreasing to 0.37 (95% CI, 0.27-0.50) per 100 PYs after ART initiation. The TB reactivation rates for those with positive LTBI test results or TB diagnoses after their HIV diagnosis are shown in eTable 3 in the Supplement.

### Base Case Model Simulation

**Figure 1.**
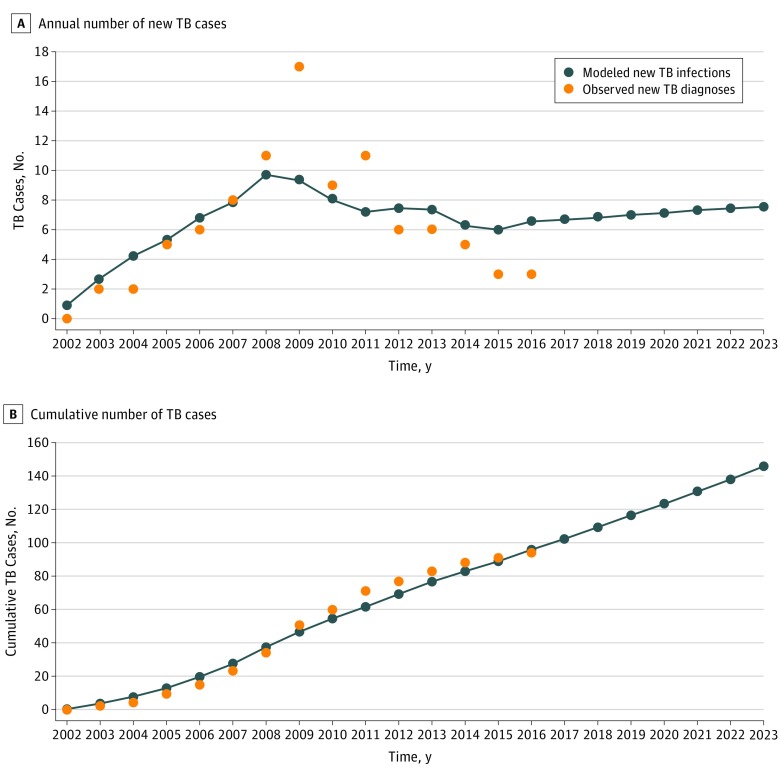
Model Simulation Results in Base Case Scenario Orange circles represent the annual number of newly diagnosed tuberculosis (TB) cases in the clinic (observed data), and the blue lines represent the annual estimated number of new TB cases in the model.

In the base case model, the cumulative estimated number of TB cases from 2002 to 2008 resembled the cumulative number of diagnosed TB cases in the clinic, despite some discrepancies after 2008 (Figure 1). Model estimates of cumulative number of TB cases would reach 146 by 2023, with the annual number of new TB diagnoses ranging from 6 to 8.

### Comparing the Effectiveness of Subsequent LTBI Testing Strategies

Comparing LTBI testing strategies, strategy D (up to 3 subsequent tests) and strategy E (annual testing) were estimated to avert the highest proportion of new TB cases above the base case scenario (Table 1), while strategy A (no subsequent testing) averted the lowest proportion of new TB cases. Comparing scenarios 1 to 5, with different coverage of ART, LTBI testing, and LTBI treatment, scenario 2 (ie, 100% coverage of ART, LTBI testing, and LTBI treatment) was estimated to avert the highest proportion of new TB cases in 2017 to 2023. This was followed by scenario 5 (100% coverage for LTBI testing and treatment only).

### Comparing the Cost-effectiveness of Subsequent LTBI Testing Strategies

Compared with no subsequent LTBI testing in each coverage scenario, offering subsequent LTBI testing by different criteria (strategies B-E) resulted in more QALYG but a higher cost. Biennial testing and current annual testing strategies were extendedly dominated, as their ICERs were higher than more effective alternative strategies. With a $50 000 per QALYG WTP threshold, no testing strategy was likely cost-effective (Table 2). If the WTP threshold were increased to $100 000 per QALYG and $200 000 per QALYG, the strategies of testing by risk factors and up to 3 subsequent tests, respectively, were likely cost-effective.

**Table 2.** Comparison of Cost-effectiveness Between Scenarios of LTBI Testing Strategies With Different Coverage Scenarios, 2017-2023

Criteria	Testing Strategy				
	A, No Subsequent LTBI Testing	B, Subsequent Annual Testing by Risk, vs A	C, Biennial Testing for All Until Positive Result, vs B	D, Up to 3 Subsequent Tests, vs B	E, Subsequent Annual Testing for All Until Positive Result, vs D
**Scenario 1: Baseline Coverage of ART, LTBI Testing, and LTBI Treatment**					
No. of new TB cases	52	51	51	50	50
No. of LTBI tests	2473	3471	5435	5791	8387
Discounted incremental cost, $	NA	20 650	40 805	48 903	50 245
Discounted QALYG	NA	0.21	0.03	0.17	0.0003
ICER, $/QALYG	NA	97 231	ED	293 371	ED
**Scenario 2: 100% Coverage of ART, LTBI Testing, LTBI Treatment, and TB Treatment**					
No. of new TB cases	33	31	31	30	30
No. of LTBI tests	2729	5221	10 210	8052	17 667
Discounted incremental cost, $	NA	51 283	97 864	51 750	187 748
Discounted QALYG	NA	0.48	0.06	0.27	0.001
ICER, $/QALYG	NA	107 583	ED	192 658	ED
**Scenario 3: 100% Coverage of ART, LTBI Treatment, and TB Treatment With Baseline Coverage of LTBI Testing**					
No. of new TB cases	46	45	44	44	44
No. of LTBI tests	2474	3448	5436	5791	8388
Discounted incremental cost, $	NA	20 192	41 070	48 677	50 245
Discounted QALYG	NA	0.21	0.04	0.19	0.0003
ICER, $/QALYG	NA	96 635	ED	260 320	ED
**Scenario 4: 100% Coverage of LTBI Treatment and TB Treatment With Baseline Coverage of ART and LTBI Testing**					
No. of new TB cases	48	47	46	46	46
No. of LTBI tests	2473	3471	5435	5791	8387
Discounted incremental cost, $	NA	20 810	40 039	47 352	50 245
Discounted QALYG	NA	0.23	0.03	0.19	0.0003
ICER, $/QALYG	NA	91 097	ED	254 157	ED
**Scenario 5: 100% Coverage of LTBI Testing, LTBI Treatment, and TB Treatment With Baseline Coverage of ART**					
No. of new TB cases	34	32	32	31	31
No. of LTBI tests	2729	5283	10 210	8052	17 668
Discounted incremental cost, $	NA	52 797	95 219	48 547	187 749
Discounted QALYG	NA	0.51	0.05	0.27	0.001
ICER, $/QALYG	NA	102 588	ED	181 710	ED

Abbreviations: ART, antiretroviral therapy; ED, extended dominance; ICER, incremental cost-effectiveness ratio; LTBI, latent tuberculosis infection; NA, not applicable; QALYG, quality-adjusted life-years gained; TB, tuberculosis.

Compared with the status quo scenario, the relative positions of LTBI testing strategies on the ICER plane were similar for different scenarios of coverage for LTBI testing and treatment (eFigure 9 in the Supplement). Strategy A was less costly but less effective, followed by higher incremental cost but more QALYG for strategies B and C. However, the relative position of strategy D depended on the coverage scenario, which would cost less in scenarios 1, 2, and 5 but more in scenarios 3 and 4 (eFigure 9 in the Supplement).

### Sensitivity Analysis

By reducing the number of newly diagnosed HIV-positive patients or LTBI prevalence while keeping other parameters constant, the discounted incremental cost dropped substantially, whereas the discounted QALYG increased compared with status quo (scenario E1) (eFigure 10A and B in the Supplement). However, compared with parameters of new HIV diagnoses and LTBI prevalence, the impact of LTBI treatment on reducing the relative proportion of TB reactivation risk on ICER was limited if there was no corresponding increase in LTBI testing coverage (eFigure 10C in the Supplement).

In the status quo scenario, increasing the coverage of LTBI testing and treatment was associated with an increase in discounted QALYG, despite a corresponding increase in discounted incremental cost (eFigure 11 in the Supplement). Compared with the status quo, applying strategies B and D under scenarios with 100% ART and TB treatment coverage, increasing LTBI testing and treatment coverage was associated with reduced discounted incremental cost but increased discounted QALYG (eFigure 12 and eFigure 13 in the Supplement). In all scenarios, increasing LTBI testing coverage was associated with a parallel shift of more QALYG with a small or negative incremental cost.

**Figure 2.**
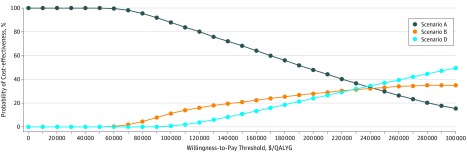
Cost-effectiveness Acceptability Curves for the Probabilistic Sensitivity Analysis Under each latent tuberculosis infection (LTBI) testing strategy (ie, A, no subsequent testing; B, subsequent test by risk factors; C, biennial testing; D, up to 3 subsequent tests; and E, annual testing), 10 000 simulations were performed for probabilistic sensitivity analysis. Random selection of values of model parameters included the prevalence of local LTBI (ie, −20% to 50% change of baseline value in 2016), LTBI treatment efficacy for reducing TB reactivation (ie, −10% to 50% change of baseline value in 2016), LTBI testing cost (ie, $12-$60), LTBI treatment and monitoring cost (ie, −30% to 30% change from baseline value in 2016), ART coverage (80%-100%), LTBI treatment coverage (ie, 60%-100%), baseline LTBI testing coverage (ie, 50%-100%), and subsequent testing coverage (ie, 40%-100%). The likelihood of strategies being cost-effective were plotted against the corresponding willingness-to-pay threshold. QALYG indicates quality-adjusted life-years gained.

In a probabilistic sensitivity analysis with 10 000 simulations, strategy A remained associated with the most cost-savings while strategies D and E were the most effective (eTable 4 in the Supplement). Strategies C and E were extendedly dominated and excluded from comparison. Taking the strategy with the highest ICER below the corresponding WTP threshold in each simulation, strategy A was the most cost-effective below the WTP threshold of $50 000 per QALYG (Figure 2). The probability of strategies B and D being the most cost-effective increased from 0% at $50 000 per QALYG to 11% and 1%, respectively, at $100 000 per QALYG. At a threshold of $240 000 per QALYG and higher, the probability of strategy D being the most cost-effective was highest.

## Discussion

In Hong Kong, a city with intermediate TB burden and low HIV coinfection incidence, our study results showed that the current strategy of annual LTBI testing for HIV-positive individuals is not cost-effective compared with less intense testing strategies. At a WTP threshold of $50 000 per QALYG, none of the 4 LTBI testing strategies (ie, annual, risk-based, biennial, and up to 3 subsequent tests) after baseline testing was likely to be cost-effective. The risk-based testing strategy and the strategy using up to 3 subsequent tests were cost-effective if the WTP threshold was increased to $100 000 per QALYG and $200 000 per QALYG, respectively. The effect of expanding LTBI treatment coverage alone was small. To be cost-effective, the concurrent expansion of coverage for both LTBI testing and treatment is essential.

In clinical practice, repeated LTBI testing is theoretically useful for identifying individuals who might have received false-negative results in the previous tests owing to defective immune status^16,27^ or those who might have become infected with TB after the first test. From our sensitivity analysis, the impact of detecting new TB infections after HIV diagnosis on the epidemic curve projection was very small. In Hong Kong, nonlocal infections (inferred from infections in non-Chinese individuals and residents without right of abode) were also not significantly associated with positive subsequent LTBI tests.^21^ To identify patients who had false-negative test results in previous LTBI tests, targeted subsequent LTBI tests by risk factor (strategy B), which cost less than annual testing strategy, might be an option. The risk-based testing strategy is currently recommended by the National Institute for Health and Care Excellence and the British HIV Association in the United Kingdom; this strategy involves testing HIV-positive individuals based on specific criteria, such as CD4 lymphocyte levels, country of origin, and ART duration.^11^ The 2 UK strategies were cost-effective compared with no testing, although the timing of LTBI testing was not stated clearly.^11^ A risk-based testing strategy has also been recommended in Belgium.^28^

Our study results support another testing strategy, strategy D, which involves repeating a small number of LTBI tests (eg, 3 subsequent tests) for HIV-positive individuals who had a negative baseline result. Currently, HIV-positive individuals begin ART within a relatively short period after diagnosis.^5^ It is possible to be receiving ART and experience immune recovery within 3 years with annual LTBI tests. If all 3 subsequent tests are negative, the chance of a false-negative result could be very low. While this strategy was effective (almost the same QALYG as the annual testing strategy), it cost more than the no testing and risk-based testing strategies. It would be cost-effective only if the WTP threshold were increased to $200 000 per QALYG. The results illustrated the small but definite benefits of targeted and time-limited LTBI following baseline screening.

Among the testing strategies, the strategy of requiring no repeated LTBI testing (strategy A) was cost-saving and likely cost-effective. The results were consistent with a US study that analyzed the cost-effectiveness of baseline LTBI testing and treatment among residents not born in the United States,^10^ showing that a confirm-negative testing strategy (ie, positive results in either initial interferon-gamma release assay or subsequent TST) was cost-effective ($63 000 per QALYG) among HIV-positive individuals. However, strategy A had the lowest QALYG among testing strategies. It was least effective in identifying a high proportion of LTBI cases followed by treatment. In clinical and public health contexts, the selection of a testing strategy requires a careful balance between effectiveness and cost-effectiveness.

A higher LTBI testing rate followed by higher treatment coverage is important for increasing effectiveness. With more testing, more LTBI cases would receive treatment, which could reduce the risk of TB reactivation substantially.^5,29,30^ In our study, 100% coverage of baseline LTBI testing and treatment without subsequent LTBI tests could avert at least 30% of new TB cases compared with the base case scenario. This highlighted the importance of the first LTBI test for reducing TB incidence, which is in line with our analysis of clinical data and a cohort study in Spain.^29^ The cost does not necessarily increase with expanded coverage for LTBI testing. Compared with the base case scenario, expanding LTBI testing coverage in strategy D (up to 3 annual tests) could decrease cost and increase QALYG, as shown in our sensitivity analyses. On the other hand, full ART coverage could avert an additional 3% to 4% of new TB infections above the base case scenario. The small marginal change was mainly because of the already high baseline ART coverage (80% of patients).

### Limitations

Our study had limitations. First, our model does not account for new TB infections occurring after HIV diagnosis. In places with higher chances of acquiring a TB infection after HIV diagnosis, the expanded model in the sensitivity analysis might be a more suitable reference (eFigure 8 in the Supplement). Second, for simplicity, we included TST, the main LTBI test in the study area, as the only LTBI test in the cost-effectiveness analysis. As only completed TSTs at the second visit were documented in the data set, the failure rate of the second visit could not be estimated. In addition, the cost borne by patients for attending the second visit has not been included, thereby underestimating the total testing cost. The additional administrative or review cost for switching to other testing strategies has not been included in cost estimation. In settings without available data for identifying patients at risk, higher related costs would need to be considered in analysis. Third, diabetes, a well-documented risk factor for TB reactivation, was not factored in the model.^31^ This was mainly because of its insignificant association with TB disease development in our analysis on the clinical data.^21^ Overall, only 4% of HIV-positive individuals in the data set had been diagnosed with diabetes. Age group and progression were included in the model structure, but age was not associated with TB reactivation risk in the model. This could again be because of the insignificant association between the 2 variables.^21^ As a short-term modeling study, the impact of age in the relatively young study population (98% of whom were younger than 65 years) is anticipated to be small. To assess the impact of risk-based testing strategy, analysis based on local empirical clinical data to examine the main risk factors of TB disease development is needed.

## Conclusions

This study suggests that the current annually repeated LTBI testing strategy for HIV-positive individuals in Hong Kong could be improved. Repeated testing is excessive because of the generally low risk of TB reactivation and infection, as shown by a high number of repeatedly negative LTBI test results. By offering a less intense subsequent LTBI testing strategy and expanding baseline LTBI testing and treatment, either risk-based testing or a limited number of yearly tests could lower cost and increase QALYG. However, the coverage of baseline LTBI testing and treatment should be increased to achieve the best health outcomes with maximum cost-effectiveness.

## Notes

Supplement. **eAppendix. **Description of the Model **eTable 1. **List of Model Parameters **eTable 2. **List of Cost and Utility **eTable 3. **TB Reactivation Rate Varied by Right of Abode, Antiretroviral Therapy Status, CD4 Level, and History of LTBI Treatment Among Patients With LTBI **eTable 4.** Incremental Cost-effectiveness Ratio of LTBI Screening Strategies Probabilistic Sensitivity Analysis **eFigure 1. **System Dynamics Model Flow Diagram and Change of States **eFigure 2. **Prevalence of LTBI Among Local and Nonlocal Patients Over Time **eFigure 3. **Proportion of Local Newly Diagnosed HIV-Positive Patients Over Time **eFigure 4. **LTBI Testing Rate Over Time **eFigure 5. **LTBI Treatment Initiation Rate Among LTBI Patients Over Time **eFigure 6. **Antiretroviral Therapy Initiation Rates **eFigure 7. **Expanded Model Structure Accounting for New TB Infection **eFigure 8. **Model Simulation Results in Expanded Model **eFigure 9. **Incremental Cost-effectiveness Ratio (ICER Plane) of LTBI Screening Strategies Under Different Coverage of Antiretroviral Therapy, LTBI Testing and Treatment, and TB Treatment, 2017-2023 **eFigure 10. **Impact of Annual Number of Newly Diagnosed HIV Cases in the Clinic, LTBI Prevalence Among HIV-Positive Patients, and Reduction of TB Reactivation by LTBI Treatment on Incremental Cost-effectiveness Ratio Under the Current Annual Testing Strategy at Baseline Coverage Value (Scenario E1) in 2017-2023 **eFigure 11. **Impact of LTBI Testing Coverage (40%-100%) and LTBI Treatment Coverage (50%-100%) Under Current Annual Testing Strategy at Baseline Coverage Value (Scenario E1) on Incremental Cost-effectiveness Ratio (ICER) in 2017-2023, 2-way Sensitivity Analysis Results **eFigure 12. **Impact of LTBI Testing Coverage (40%-100%) and LTBI Treatment Coverage (50%-100%) Under at Most 3 Subsequent LTBI Testing Strategy (Scenario D) with 100% Antiretroviral Therapy Coverage on Incremental Cost-effectiveness Ratio (ICER) in 2017-2023, 2-way Sensitivity Analysis Results **eFigure 13. **Impact of LTBI Testing Coverage (40%-100%) and LTBI Treatment Coverage (50%-100%) Under Strategy of Testing by Risk Factor (Scenario B) With 100% Antiretroviral Therapy Coverage on Incremental Cost-effectiveness Ratio (ICER) in 2017-2023, 2-way Sensitivity Analysis Results **eReferences.**
