# Supplementary material for: Latent Tuberculosis Infection Testing Strategies for HIV-Positive Individuals in Hong Kong
Source: JAMA Netw Open. 2019 Sep 6;2(9):e1910960. doi: 10.1001/jamanetworkopen.2019.10960 (PMC6735410; doi:10.1001/jamanetworkopen.2019.10960)
Supplement: Supplement. — eAppendix. Description of the Model eTable 1. List of Model Parameters eTable 2. List of Cost and Utility eTable 3. TB Reactivation Rate Varied by Right of Abode, Antiretroviral Therapy Status, CD4 Level, and History of LTBI Treatment Among Patients With LTBI eTable 4. Incremental Cost-effectiveness Ratio of LTBI Screening Strategies Probabilistic Sensitivity Analysis eFigure 1. System Dynamics Model Flow Diagram and Change of States eFigure 2. Prevalence of LTBI Among Local and Nonlocal Patients Over Time eFigure 3. Proportion of Local Newly Diagnosed HIV-Positive Patients Over Time eFigure 4. LTBI Testing Rate Over Time eFigure 5. LTBI Treatment Initiation Rate Among LTBI Patients Over Time eFigure 6. Antiretroviral Therapy Initiation Rates eFigure 7. Expanded Model Structure Accounting for New TB Infection eFigure 8. Model Simulation Results in Expanded Model eFigure 9. Incremental Cost-effectiveness Ratio (ICER Plane) of LTBI Screening Strategies Under Different Coverage of Antiretroviral Therapy, LTBI Testing and Treatment, and TB Treatment, 2017-2023 eFigure 10. Impact of Annual Number of Newly Diagnosed HIV Cases in the Clinic, LTBI Prevalence Among HIV-Positive Patients, and Reduction of TB Reactivation by LTBI Treatment on Incremental Cost-effectiveness Ratio Under the Current Annual Testing Strategy at Baseline Coverage Value (Scenario E1) in 2017-2023 eFigure 11. Impact of LTBI Testing Coverage (40%-100%) and LTBI Treatment Coverage (50%-100%) Under Current Annual Testing Strategy at Baseline Coverage Value (Scenario E1) on Incremental Cost-effectiveness Ratio (ICER) in 2017-2023, 2-way Sensitivity Analysis Results eFigure 12. Impact of LTBI Testing Coverage (40%-100%) and LTBI Treatment Coverage (50%-100%) Under at Most 3 Subsequent LTBI Testing Strategy (Scenario D) with 100% Antiretroviral Therapy Coverage on Incremental Cost-effectiveness Ratio (ICER) in 2017-2023, 2-way Sensitivity Analysis Results eFigure 13. Impact of LTBI Testing Coverage (40%-100 [file jamanetwopen-2-e1910960-s001.pdf]

Supplementary Online Content

Wong NS, Chan KCW, Wong BCK, et al. Latent tuberculosis infection testing strategies for HIV-positive individuals in Hong Kong. *JAMA Netw Open*. 2019;2(9):1910960. doi:10.1001/jamanetworkopen.2019.10960

eAppendix. Description of the Model
eTable 1 List of Model Parameters
eTable 2. List of Cost and Utility
eTable 3. TB Reactivation Rate Varied by Right of Abode, Antiretroviral Therapy Status, CD4 Level, and History of LTBI Treatment Among Patients With LTBI
eTable 4. Incremental Cost-Effectiveness Ratio of LTBI Screening Strategies Probabilistic Sensitivity Analysis
eFigure 1. System Dynamics Model Flow Diagram and Change of States
eFigure 2. Prevalence of LTBI Among Local and Nonlocal Patients Over Time
eFigure 3. Proportion of Local Newly Diagnosed HIV-Positive Patients Over Time
eFigure 4. LTBI Testing Rate Over Time
eFigure 5. LTBI Treatment Initiation Rate Among LTBI Patients Over Time
eFigure 6. Antiretroviral Therapy Initiation Rates
eFigure 7. Expanded Model Structure Accounting for New TB Infection
eFigure 8. Model Simulation Results in Expanded Model
eFigure 9. Incremental Cost-Effectiveness Ratio (ICER Plane) of LTBI Screening Strategies Under Different Coverage of Antiretroviral Therapy, LTBI Testing and Treatment, and TB Treatment, 2017-2023
eFigure 10. Impact of Annual Number of Newly Diagnosed HIV Cases in the Clinic, LTBI Prevalence Among HIV-Positive Patients, and Reduction of TB Reactivation by LTBI Treatment on Incremental Cost-Effectiveness Ratio Under the Current Annual Testing Strategy at Baseline Coverage Value (Scenario E1) in 2017-2023
eFigure 11. Impact of LTBI Testing Coverage (40%-100%) and LTBI Treatment Coverage (50%-100%) Under Current Annual Testing Strategy at Baseline Coverage Value (Scenario E1) on Incremental Cost-Effectiveness Ratio (ICER) in 2017-2023, 2-way Sensitivity Analysis Results
eFigure 12. Impact of LTBI Testing Coverage (40%-100%) and LTBI Treatment Coverage (50%-100%) Under at Most 3 Subsequent LTBI Testing Strategy (Scenario D) with 100% Antiretroviral Therapy Coverage on Incremental Cost-Effectiveness Ratio (ICER) in 2017-2023, 2-way Sensitivity Analysis Results
eFigure 13. Impact of LTBI Testing Coverage (40%-100%) and LTBI Treatment Coverage (50%-100%) Under Strategy of Testing by Risk Factor (Scenario B) With 100% Antiretroviral Therapy Coverage on Incremental Cost-Effectiveness Ratio (ICER) in 2017-2023, 2-way Sensitivity Analysis Results
eReferences.

This supplementary material has been provided by the authors to give readers additional information about their work.

© 2019 Wong NS et al. *JAMA Network Open.*

eAppendix. Description of the Model

In the model, we classified the study population by 24 states based on age group, CD4 level, antiretroviral therapy (ART) status, and right of abode (non-local, local); young (aged 18-49), middle-aged (aged 50-64), older aged (aged >64); pre-ART CD4 ≥200/µl, pre-ART CD4 <200/µl, on ART CD4 ≥200/µl, on ART CD4 <200/µl):

Non-local, pre-ART:
young and high CD4 (i=1); young and low CD4 (i=2); middle-aged and high CD4 (i=3); middle-aged and low CD4 (i=4); older-aged and high CD4 (i=5); older-aged and low CD4 (i=6);

Local, pre-ART:
young and high CD4 (i=7); young and low CD4 (i=8); middle-aged and high CD4 (i=9); middle-aged and low CD4 (i=10); older-aged and high CD4 (i=11); older-aged and low CD4 (i=12);

Non-local, on ART:
young and high CD4 (i=13); young and low CD4 (i=14); middle-aged and high CD4 (i=15); middle-aged and low CD4 (i=16); older-aged and high CD4 (i=17); older-aged and low CD4 (i=18);

Local, on ART:
young and high CD4 (i=19); young and low CD4 (i=20); middle-aged and high CD4 (i=21); middle-aged and low CD4 (i=22); older-aged and high CD4 (i=23); older-aged and low CD4 (i=24);

Over time, study populations progressed into older age groups, experienced CD4 decline before ART initiation, received ART, and experienced CD4 recovery after ART initiation. The rate was estimated from empirical clinical dataset of LTBI infected patients diagnosed with HIV between 2002 and 2013, with follow up time through mid-2017 (**eTable 1**).

CD4 200/µl is used as a threshold for defining states. In logistic regression model, the odds ratio (OR) between positive latent TB infection (LTBI) test results (defined as >5mm induration in tuberculin sensitivity testing) and CD4≥100/µL was 14, CD4≥200/µL was 3.02, CD4≥300/µL was 2.41, and CD4≥400/µL was 2.64.

Model equations

Time step = month

For HIV patients without LTBI

Waiting for the first LTBI test (never testers)

$$\frac{dNegLTBI_i}{dt} = newdx[t,i] * \left(plocal[t,i] * (1 - pLTBIlocal[t,i]) + (1 - plocal[t,i]) * (1 - pLTBInlocal[t,i])\right) + changestate[i] * NegLTBI[t,k] - (LTBIbasetest[t] + m) * NegLTBI[t,i];$$

where **newdx[t,i]** refers to annual number of newly diagnosed HIV-infected individuals of each state (i); **plocal** refers to proportion of local HIV patients per year; **pLTBIlocal** refers to LTBI prevalence among local HIV patients per year; **pLTBInlocal** refers to LTBI prevalence among non-local HIV patients per year; **LTBIbasetest** refers to baseline LTBI screening rate per year; **changestate[i]** refers to rate of changing between states (k) (Supplementary Table 1), for instance, changing from young non-local pre-ART high CD4 to low CD4 would be cd4e*compartment[i=1]; **m** refers to overall mortality rate;

Available for the first followup LTBI test following baseline LTBI test

$$\frac{dNegLTBIF1_i}{dt} = LTBIbasetest[t] * NegLTBI[t,i] + changestate[i] * NegLTBIF1[t,k] - (LTBIfltest1[t,i] + m) * NegLTBIF1[t,i];$$

where **LTBItest1[t,i]** refers to rate of subsequent LTBI test per year, and the testing rate is basically the same for all states[i]. However, it was different across states under the scenario of testing by risk factor strategy.

Available for the second followup LTBI test following the first followup LTBI test

$$\frac{dNegLTBIF2_i}{dt} = LTBIfltest1[t,i] * NegLTBIF1[t,i] + changestate[i] * NegLTBIF2[t,k] - (LTBIfltest2[t,i] + m) * NegLTBIF2[t,i];$$

© 2019 Wong NS et al. *JAMA Network Open.*

where **LTBItest2[t,i]** refers to rate of subsequent LTBI test per year, and it is basically the same as LTBItest1[t,i], except under scenarios of no subsequent LTBI test.

Available for the third followup LTBI test following the second followup LTBI test

$$\frac{dNegLTBIF3_i}{dt} = LTBIfltest2[t,i] * NegLTBIF2[t,i] + changestate[i] * NegLTBIF3[t,k] - (LTBIfltest3[t,i] + m) * NegLTBIF3[t,i];$$

where **LTBItest3[t,i]** refers to rate of subsequent LTBI test per year, and it is basically the same as LTBItest1[t,i], except under scenarios of no subsequent LTBI test.

Available for the fourth or more followup LTBI test following the third followup LTBI test

$$\frac{dNegLTBIF4_i}{dt} = LTBIfltest3[t,i] * NegLTBIF3[t,i] + changestate[i] * NegLTBIF4[t,k] - m * NegLTBIF4[t,i];$$

LTBI test counter

$$\frac{dNegtestcount_i}{dt} = LTBIbasetest[t] * \sum_{i=1}^{24} NegLTBI[t,i] + \sum_{i=1}^{24}(LTBIfltest1[t,i] * NegLTBIF1[t,i]) + \sum_{i=1}^{24}(LTBIfltest2[t,i] * NegLTBIF2[t,i]) + \sum_{i=1}^{24}(LTBIfltest3[t,i] * NegLTBIF3[t,i]) + \sum_{i=1}^{24}(LTBIfltest4[t,i] * NegLTBIF4[t,i]);$$

where **LTBItest4[t,i]** refers to rate of subsequent LTBI test per year, and it is basically the same as LTBItest1[t,i], except under scenarios of no subsequent LTBI test and at most three subsequent LTBI tests.

For HIV patients with LTBI

Waiting for the first LTBI test (never testers)

$$\frac{dLTBI_i}{dt} = newdx[t,i] * ((plocal[t,i] * (pLTBIlocal[t,i]) + (1 - plocal[t,i]) * pLTBInlocal[t,i]) + changestate[i] * LTBI[t,k] - (LTBIbasetest[t] + (1 - LTBIbasetest[t]) * TBdxntest[i] + m) * LTBI[t,i];$$

where **TBdxntest[i]** refers to TB reactivation rate for never testers with LTBI;

Tested baseline LTBI positive, without LTBI treatment

$$\frac{dLTBI\_NT_i}{dt} = LTBIbasetest[t] * LTBIposb[i] * LTBI[t,i] + changestate[i] * LTBI\_NT[t,k] - (TBdxb[i] + LTBItx[t] + m) * LTBI\_NT[t,i];$$

where **LTBIposb[i]** refers to the positive rate of LTBI test; **TBdxb[i]** refers to TB reactivation rate for patients tested LTBI positive but not yet received LTBI treatment, and at baseline, TBdxb[i] was the same as TBdxntest[i]; **LTBItx[t]** refers to LTBI treatment initiation rate;

Tested baseline LTBI positive, received LTBI treatment

$$\frac{dLTBI\_T_i}{dt} = LTBItx[t] * LTBI\_NT[t,i] + changestate[i] * LTBI\_T[t,k] - (TBdxb\_tx[i] + m) * LTBI\_T[t,i];$$

where **TBdxb_tx[i]** refers to TB reactivation rate for patients tested LTBI positive received LTBI treatment;

Available for the first followup LTBI test following negative baseline LTBI test result

© 2019 Wong NS et al. *JAMA Network Open.*

$$\frac{dLTBI\_F1_i}{dt} = LTBIbasetest[t] * (1 - LTIBposb[i]) * LTBI[t,i] + changestate[i] * LTBI\_F1[t,k] - (LTBIfltest1[t,i] + (1 - LTBIfltest1[t,i]) * TBdxntestf[i] + m) * LTBI\_F1[t,i];$$

where **TBdxntestf[i]** refers to TB reactivation rate for patients not yet tested LTBI positive at subsequent test, and at baseline**, TBdxntestf[i]** was the same as TBdxntest[i];

Available for the second followup LTBI test following negative result at the first followup LTBI test

$$\frac{dLTBI\_F2_i}{dt} = LTBIfltest1[t,i] * (1 - LTIBposf[i]) * LTBI\_F1[t,i] + changestate[i] * LTBI\_F2[t,k] - (LTBIfltest2[t,i] + (1 - LTBIfltest2[t,i]) * TBdxntestf[i] + m) * LTBI\_F2[t,i];$$

where **LTBIposf[i]** refers to the positive rate of subsequent LTBI test, same as LTBIposb[i];

Available for the third followup LTBI test following negative result at the second followup LTBI test

$$\frac{dLTBI\_F3_i}{dt} = LTBIfltest2[t,i] * (1 - LTIBposf[i]) * LTBI\_F2[t,i] + changestate[i] * LTBI\_F3[t,k] - (LTBIfltest3[t,i] + (1 - LTBIfltest3[t,i]) * TBdxntestf[i] + m) * LTBI\_F3[t,i];$$

Available for the fourth or more followup LTBI test following negative result at the third followup LTBI test

$$\frac{dLTBI\_F4_i}{dt} = LTBIfltest3[t,i] * (1 - LTIBposf[i]) * LTBI\_F3[t,i] + changestate[i] * LTBI\_F4[t,k] - (LTBIfltest4[t,i] * LTBIposf[i] + (1 - LTBIfltest4[t,i]) * TBdxntestf[i] + m) * LTBI\_F4[t,i];$$

Tested LTBI positive at followups, without LTBI treatment

$$\frac{dLTBI\_FNT_i}{dt} = LTBIposf[i] * (LTBIfltest1[t,i] * LTBI\_F1[t,i] + LTBIfltest2[t,i] * LTBI\_F2[t,i] + LTBIfltest3[t,i] * LTBI\_F3[t,i] + LTBIfltest4[t,i] * LTBI\_F4[t,i]) + changestate[i] * LTBI\_FNT[t,k] - (TBdxf[i] + LTBItx[t] + m) * LTBI\_FNT[t,i];$$

where **TBdxf[i]** refers to TB reactivation rate for patients tested LTBI positive but not yet received LTBI treatment, and at baseline, it was the same as TBdxntest[i];

Tested LTBI positive at followups, received LTBI treatment

$$\frac{dLTBI\_FT_i}{dt} = LTBItx[t] * LTBI\_FNT[t,i] + changestate[i] * LTBI\_FT[t,k] - (TBdxf\_tx[i] + m) * LTBI\_FT[t,i];$$

where **TBdxf_tx[i]** refers to TB reactivation rate for patients tested LTBI positive received LTBI treatment, and at baseline, it was the same as TBdxb_tx[i];

LTBI test counter

$$\frac{dtestcount_i}{dt} = LTBIbasetest[t] * \sum_{i=1}^{24} LTBI[t,i] + \sum_{i=1}^{24}(LTBIfltest1[t,i] * LTBI\_F1[t,i]) + \sum_{i=1}^{24}(LTBIfltest2[t,i] * LTBI\_F2[t,i]) + \sum_{i=1}^{24}(LTBIfltest3[t,i] * LTBI\_F3[t,i]) + \sum_{i=1}^{24}(LTBIfltest4[t,i] * LTBI\_F4[t,i]);$$

TB cases

$$\frac{dTB\_dx}{dt} = (1 - LTBIbasetest[t]) * \sum_{i=1}^{24}(TBdxntest[i] * LTBI[t,i]) + \sum_{i=1}^{24}\left(TBdxb[i] * LTBI_{NT[t,i]}\right)$$
$$+ \sum_{i=1}^{24}\left(TBdxb_{tx[i]} * LTBI_{T[t,i]}\right)$$
$$+ \sum_{i=1}^{24}((1 - LTBIfltest1[t,i]) * TBdxntestf[i] * LTBI\_F1[t,i])$$
$$+ \sum_{i=1}^{24}((1 - LTBIfltest2[t,i]) * TBdxntestf[i] * LTBI\_F2[t,i])$$
$$+ \sum_{i=1}^{24}((1 - LTBIfltest3[t,i]) * TBdxntestf[i] * LTBI\_F3[t,i])$$
$$+ \sum_{i=1}^{24}((1 - LTBIfltest4[t,i]) * TBdxntestf[i] * LTBI\_F4[t,i])$$
$$+ \sum_{i=1}^{24}(TBdxf[i] * LTBI\_FNT[t,i]) + \sum_{i=1}^{24} TBdxf\_tx[i] * LTBI\_FT[t,i]$$
$$- (m + mt + TBRxrate[t]) * TBdx[t];$$

© 2019 Wong NS et al. *JAMA Network Open.*

eTable 1 List of Model Parameters

Parameter	Notation	Value, range	calculation	Reference
**Characteristics of HIV patients**				
LTBI prevalence among non-local HIV patients per year	pLTBInlocal[t]	It ranges between 29% and 57% in 2002-2013. (**eFigure 2**)	No. of non-local LTBI / total no. of non-local dx in the year	Clinical dataset
LTBI prevalence among local HIV patients per year	pLTBIlocal[t]	It ranges between 22% and 47% in 2002-2013. (**eFigure 2**)	No. of local LTBI / total no. of local dx in the year	Clinical dataset
% of non-local HIV patients per year	1-plocal[t]	It ranges between 75% and 95% in 2002-2013. (**eFigure 3**)	No. of non-local HIV patients / total no. of HIV patients dx in the year	Clinical dataset
Annual number of newly diagnosed HIV patients by states:	newdx[t, i]		No. of newly diagnosed patients of states 1-12 per year	Clinical dataset
**Non-local, pre-ART:**				
young and high CD4	i=1	7-25		
young and low CD4	i=2	0-17		
middle-aged and high CD4	i=3	0-1		
middle-aged and low CD4	i=4	0-1		
older-aged and high CD4	i=5	0		
older-aged and low CD4	i=6	0		
**Local, pre-ART**				
young and high CD4	i=7	46-220		
young and low CD4	i=8	20-57		
middle-aged and high CD4	i=9	1-17		
middle-aged and low CD4	i=10	2-13		
older-aged and high CD4	i=11	0-4		
older-aged and low CD4	i=12	0-6		
**Change of status**				
Interval from CD4 decline (pre-ART) from	cd4e	62 months		[1]

© 2019 Wong NS et al. *JAMA Network Open.*

≥200/μL to below 200/μL				
Interval CD4 recovery (on-ART) from <200/μL to 200/μL or above	cd4f	1 year (ranging 1 month to 2 years)		[2]
Interval from young (18-49) to middle-aged (50-64)	agec	31 years		
Interval from middle-aged to older aged (>64)	aged	14 years		
ART initiation rate among patients with CD4 ≥200/μL per year	Txg_n[t], Txg[t]	It ranges between 0% and100% in 2002-2016 among patients never tested for LTBI (Txg_n[t]), and ranges between 0% and 65% among patients ever tested for LTBI (Txg[t]).	No. of ART initiation / no. of patients available for ART initiation, CD4≥200/μL	Clinical dataset
ART initiation rate among patients with CD4 <200/μL per year	Txh_n[t], Txh[t]	It ranges between 0% and 100% in 2002-2016 among patients never tested for LTBI(Txh_n[t]) and ever tested for LTBI (Txh[t]).	No. of ART initiation / no. of patients available for ART initiation, CD4<200/μL	Clinical dataset
**LTBI testing**				
Baseline LTBI screening rate per year	LTBIbasetest [t]	It ranges between 44% and 64% in 2002-2016. (**eFigure 4**)	No. of first LTBI tester/ total no. of patients available for first LTBI test in the year, among LTBI	Clinical dataset
Subsequent LTBI screening rate per year	LTBIfltest[t], LTBItest1[t], LTBItest2[t], LTBItest3[t], LTBItest4[t]	It ranges between 39% and 66% in 2002-2016. (**eFigure 4**)	No. of followup test / total no. of patients available for followup test in the year, among LTBI	Clinical dataset
positive rate of LTBI test by states in patients with LTBI	LTBIposb[i], LTBIposf[i]	It ranges between 71% and 100% across 24 states.	No. of positive LTBI screened/ total no. of LTBI tests in the year	Clinical dataset
**TB reactivation rate (by LTBI treatment history, locality, pre-ART and on ART CD4 level)**	TBdxb[i], TBdxf[i], TBdxb_tx [i], TBdxf_tx[i], TBdxntest[i], TBdxntestf[i]	TB reactivation per 100 person-year among LTBI patients who were diagnosed with HIV in 2002-2013 (**eTable 3**).		Clinical dataset

© 2019 Wong NS et al. *JAMA Network Open.*

**LTBI treatment initiation rate**	LTBItx[t]	It ranges between 44% and 76% in 2002-2016. (**eFigure 5**)	No. of LTBI treatment / total no. of patients available for followup test in the year, among LTBI	Clinical dataset
**TB treatment initiation rate**	TBRxrate	36/94=0.383		Clinical dataset
Overall mortality rate among HIV patients	m	54/19605=0.0028		Clinical dataset
Additional TB-related mortality rate	mt	2/19605=0.0001		Clinical dataset
Proportion of MDR-TB among TB-HIV cases		1.6%		[3]

[t] refers to the time-steps (by month), [i] refers to 24 states;
ART – antiretroviral therapy; LTBI – latent tuberculosis infection; TB – tuberculosis;

© 2019 Wong NS et al. *JAMA Network Open.*

eTable 2. List of Cost and Utility

Parameter	Value/calculation	Reference
**Cost (in USD)**		
Tuberculin skin test	Material cost: USD8.9/test<br>Staff cost: USD10.85/test (20 mins in 1st visit, and 10 mins in 2nd visit)<br>Total: USD19.8	Tubersol Tuberculin Purified Protein Derivative PPD (1ml) 10 Test Vial (Mantoux)<br>Retail Price: $89.42 for 10 http://www.egeneralmedical.com/mme-10733.html
TB screening for those tested LTBI positive	USD191	Items for TB screening with reference to local TB manual [4]
LTBI treatment and monitoring cost	USD316 for 9-month	[5]
TB treatment and monitoring cost	USD10,720 for 6-month	TB treatment regimen with reference to local TB manual [4]
MDR-TB treatment and monitoring cost	USD92,797 for 18-month	MDR-TB treatment regimen with reference to local TB manual [4]
**Utility**		
without TB	1	
Active TB with CD4≥200/μL	0.83	[6]
Active TB with CD4<200/μL	0.702	[6]
MDR-TB	0.68	[7]
Death	0	

Mortality rate = no. of deaths / follow-up years (from HIV dx to data collection end point or date of death, assuming complete death records even among those lost to follow-up)

© 2019 Wong NS et al. *JAMA Network Open.*

eTable 3. TB Reactivation Rate Varied by Right of Abode, Antiretroviral Therapy Status, CD4 Level, and History of LTBI Treatment Among Patients With LTBI

pre-ART						On ART					
**Right of abode (local)**	**LTBI Rx**	**pre-ART CD4 >=200**	**follow-up yrs**	**TB cases**	**TB reactivation rate (no. of TB cases/followup PY)**	**local**	**LTBI Rx**	**ART CD4 >=200**	**follow-up yrs**	**TB cases**	**TB reactivation rate (no. of TB cases/followup PY)**
**0**	**0**	**0**	23	9	0.3913	**0**	**0**	**0**	7	3	0.4286
**0**	**0**	**1**	43	9	0.2093	**0**	**0**	**1**	11	1	0.0909
**0**	**1**	**0**	25	0	0.0000	**0**	**1**	**0**	51	1	0.0196
**0**	**1**	**1**	54	0	0.0000	**0**	**1**	**1**	45	0	0.0000
**1**	**0**	**0**	103	11	0.1068	**1**	**0**	**0**	241	23	0.0954
**1**	**0**	**1**	139	11	0.0791	**1**	**0**	**1**	216	5	0.0231
**1**	**1**	**0**	390	2	0.0051	**1**	**1**	**0**	1665	3	0.0018
**1**	**1**	**1**	697	0	0.0000	**1**	**1**	**1**	1591	4	0.0025

ART – antiretroviral therapy; LTBI – latent tuberculosis infection; PY – person-years; Rx – treatment; TB – tuberculosis;

© 2019 Wong NS et al. *JAMA Network Open.*

eTable 4. Incremental Cost-Effectiveness Ratio of LTBI Screening Strategies Probabilistic Sensitivity Analysis

Under each LTBI testing strategy, 10,000 simulations were performed for probabilistic sensitivity analysis (PSA). Random selection of values of model parameters included the prevalence of local LTBI (-20% to 50% change of baseline value), LTBI treatment efficacy for reducing TB reactivation (-10% to 50% change of baseline value), LTBI testing cost (USD12-60), LTBI treatment and monitoring cost (-30% to 30% change from baseline value), ART coverage (80-100%), LTBI treatment coverage (60-100%), baseline LTBI testing coverage (50-100%) and subsequent testing coverage (40% - 100%).

	**Subsequent LTBI testing strategies (for patients tested negative at baseline LTBI screening)**				
	**A - no test**	**B - test by risk**	**C - biennial testing**	**D - up to three tests**	**E - annual test**
median incremental cost (USD) (2.5%~97.5%)	*ref*	57501 (19823~124508)	169668 (58118~367957)	155043 (60384~274408)	334344 (112610~729348)
median QALYG (2.5%~97.5%)		0.3 (0.18~0.46)	0.35 (0.23~0.51)	0.51 (0.35~0.72)	0.51 (0.35~0.72)
median ICER (2.5%~97.5%)		194858 (73439~395978)	494361 (186988~953610)	301864 (113553~572684)	660958 (239229~1343378)
median incremental cost (USD) (2.5%~97.5%)	/	*ref*	112183 (38281~243221)	95462 (36866~160225)	276724 (92498~604901)
median QALYG (2.5%~97.5%)			0.05 (0.02~0.07)	0.21 (0.17~0.26)	0.21 (0.17~0.26)
median ICER (2.5%~97.5%)			2385912 (859588~4954963)	446154 (166223~824020)	1310024 (465483~2706382)

ICER – Incremental cost-effectiveness ratio; LTBI – latent TB infection; QALYG – quality-adjusted life-years gained

© 2019 Wong NS et al. *JAMA Network Open.*

eFigure 1. System Dynamics Model Flow Diagram and Change of States

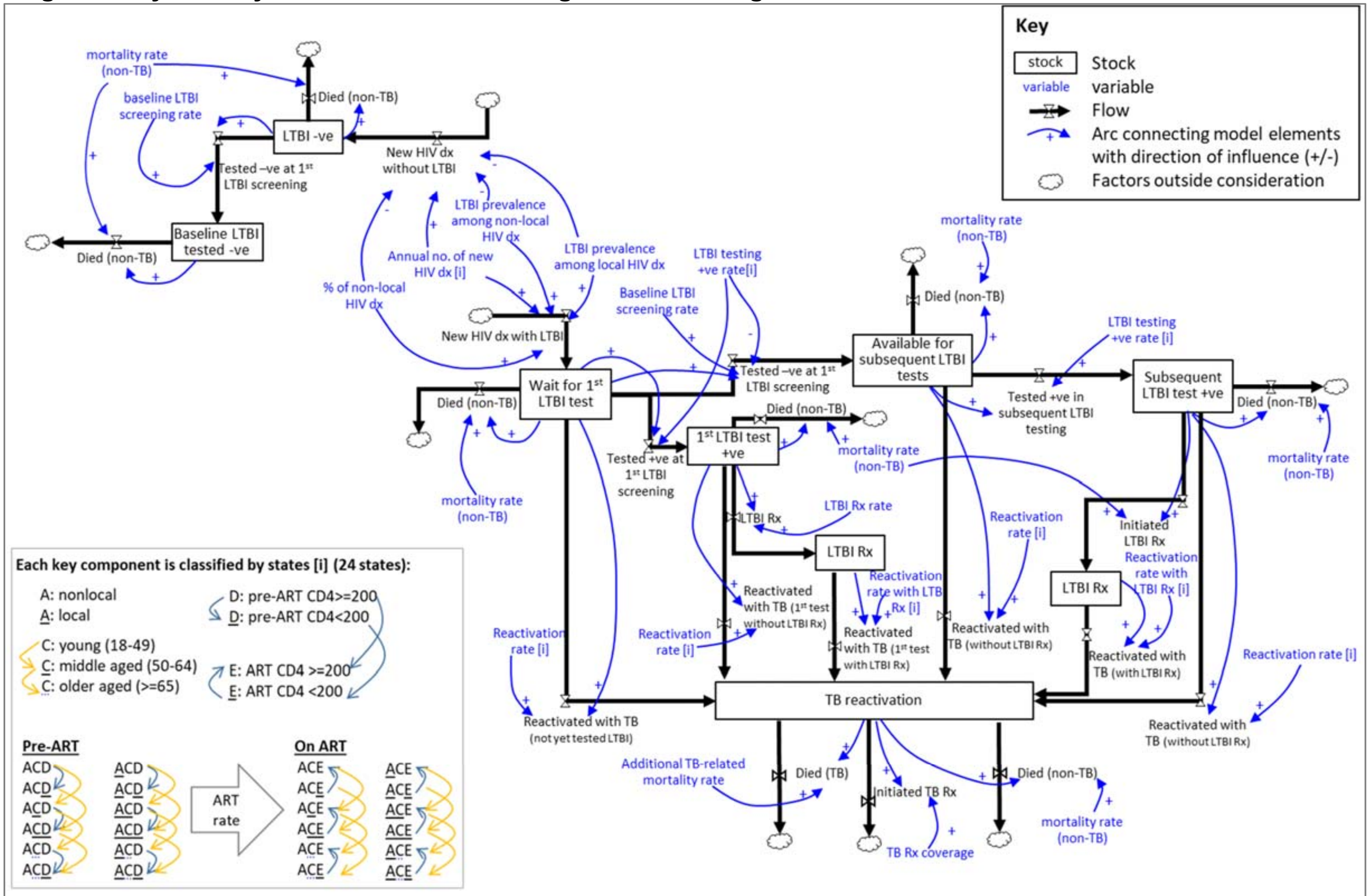

-ve: negative; ART: antiretroviral therapy; dx: diagnosis; [i]: states of subpopulations by age group, ART status, CD4 level and right of abode (nonlocal); LTBI: latent TB infection; Rx: treatment;

© 2019 Wong NS et al. *JAMA Network Open.*

eFigure 2. Prevalence of LTBI Among Local and Nonlocal Patients Over Time

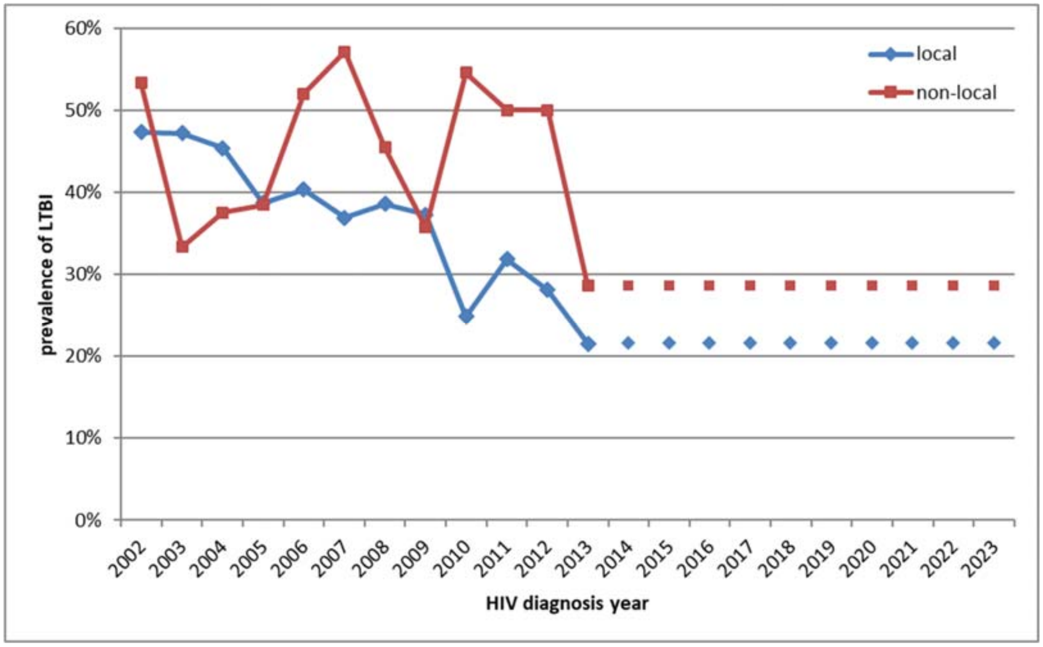

© 2019 Wong NS et al. *JAMA Network Open.*

eFigure 3. Proportion of Local Newly Diagnosed HIV-Positive Patients Over Time

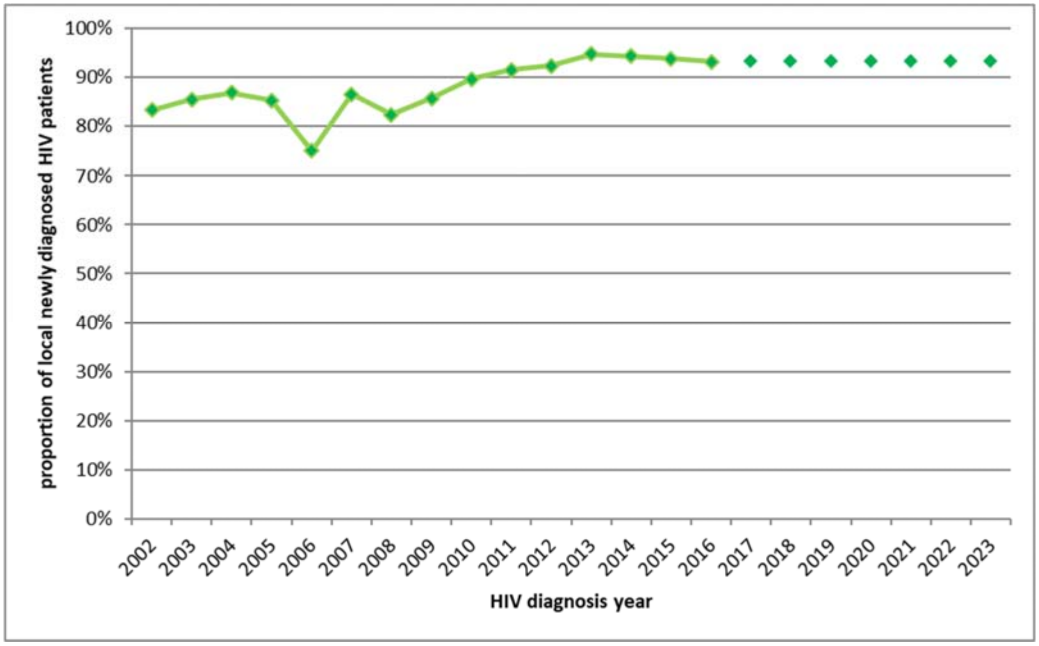

© 2019 Wong NS et al. *JAMA Network Open.*

eFigure 4. LTBI Testing Rate Over Time

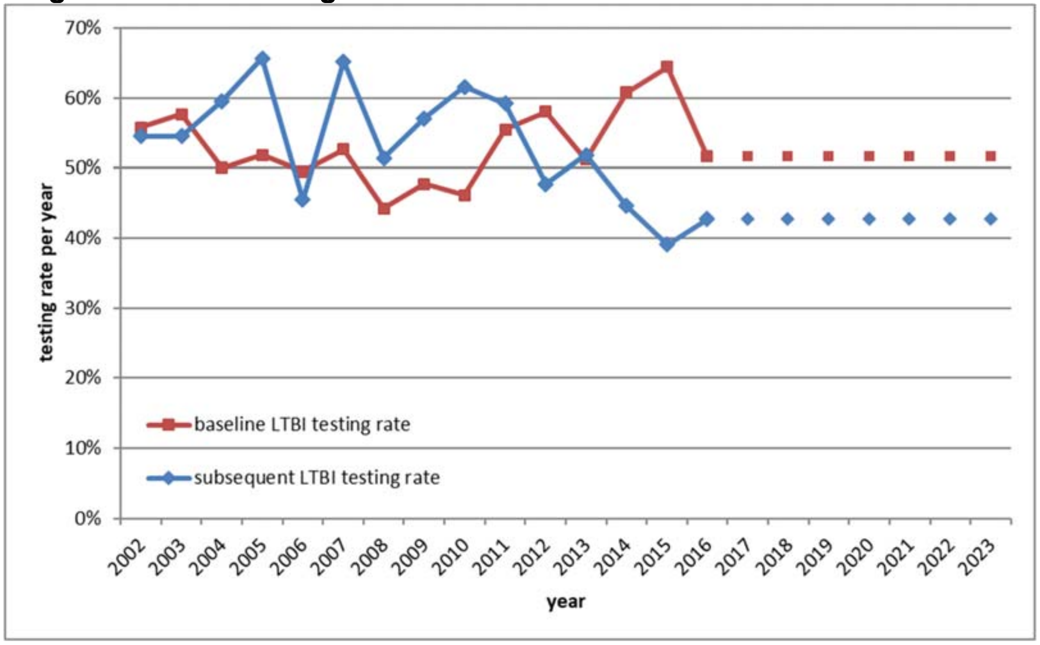

© 2019 Wong NS et al. *JAMA Network Open.*

eFigure 5. LTBI Treatment Initiation Rate Among LTBI Patients Over Time

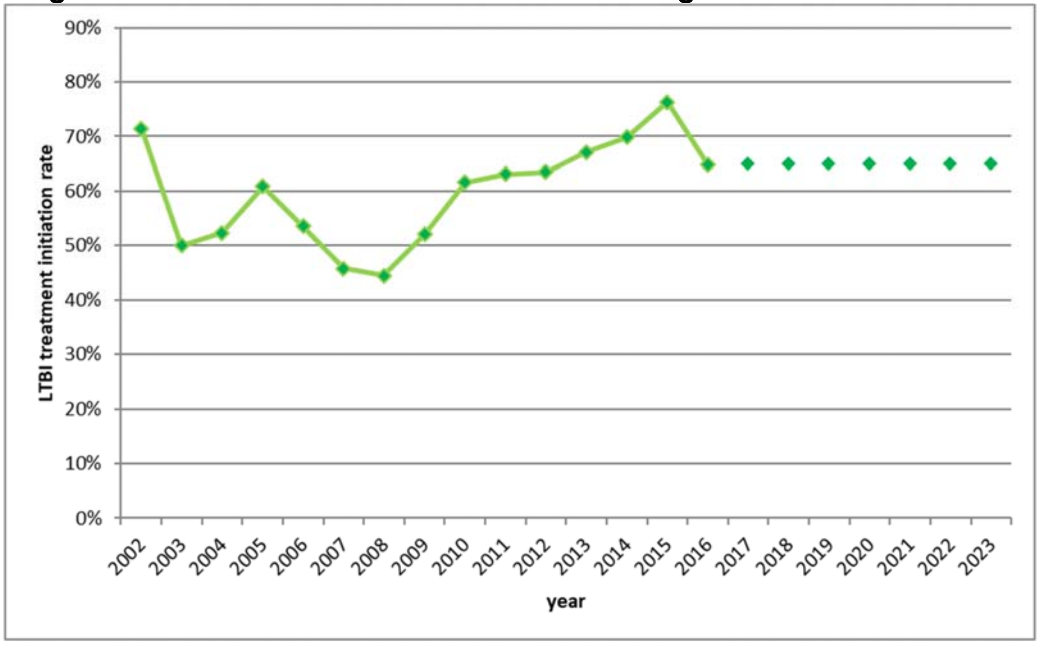

© 2019 Wong NS et al. *JAMA Network Open.*

eFigure 6. Antiretroviral Therapy Initiation Rates

A. Antiretroviral therapy initiation rate per year by CD4 level (<200/μL and ≥200/μL)

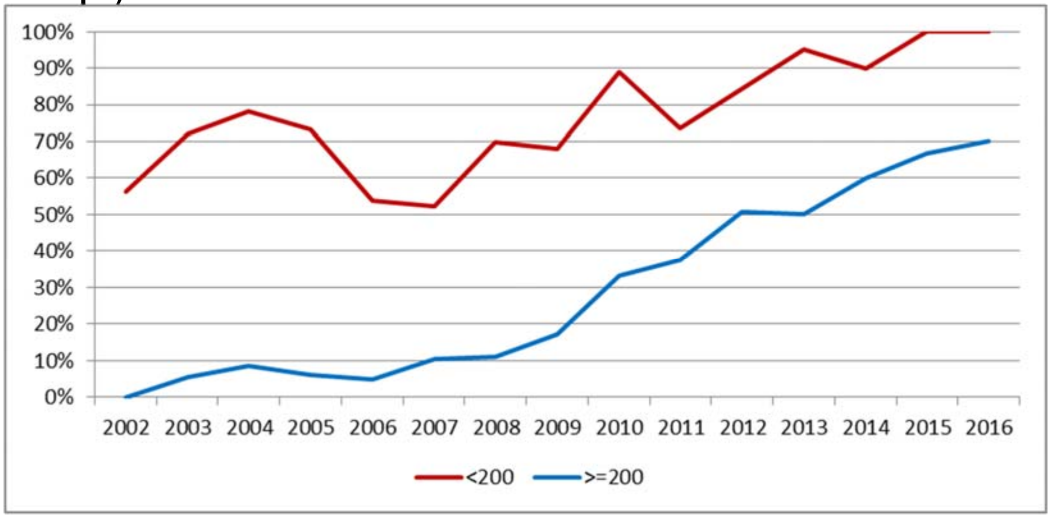

B. Antiretroviral therapy initiation rate per year by CD4 level (<200/μL and ≥200/μL) and history of LTBI test (never testers and ever testers)

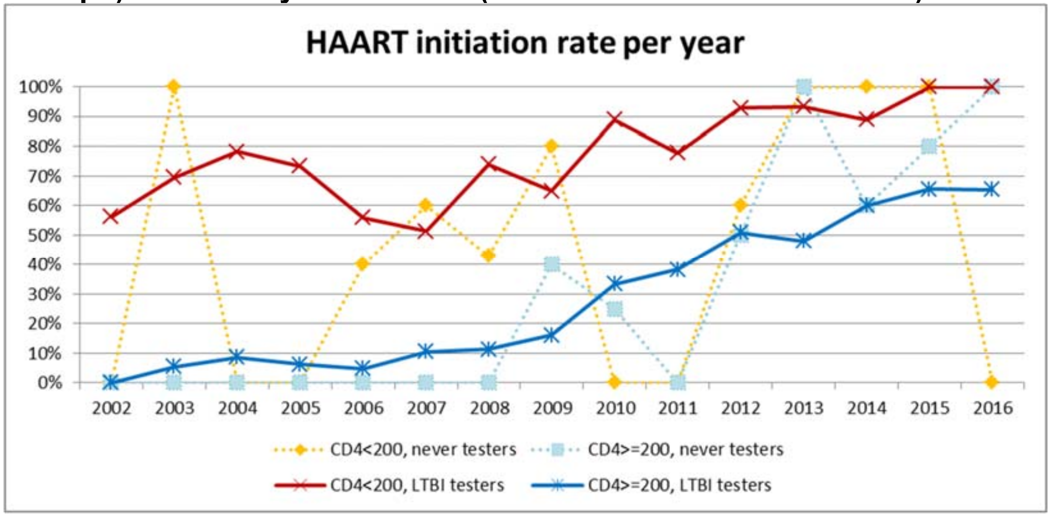

© 2019 Wong NS et al. *JAMA Network Open.*

eFigure 7. Expanded Model Structure Accounting for New TB Infection

Possible new TB infections after HIV diagnosis and baseline LTBI testing are added, with the flow highlighted in red

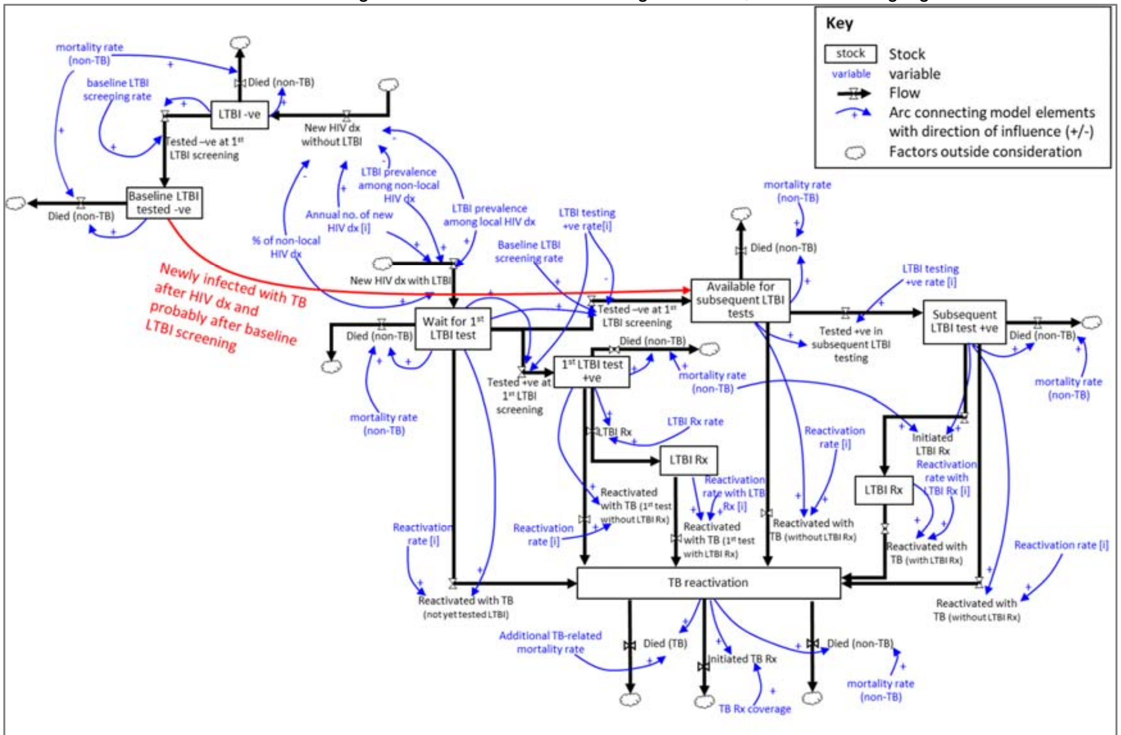

-ve: negative; ART: antiretroviral therapy; dx: diagnosis; [i]: states of subpopulations by age group, ART status, CD4 level and right of abode (nonlocal); LTBI: latent TB infection; Rx: treatment

eFigure 8. Model Simulation Results in Expanded Model

Comparison between simulation results in basecase model with the assumption of no new TB infection after HIV diagnosis (blue line) and new structure model with the inclusion of new TB infections after HIV diagnosis (green line), with reference to cumulative number of TB diagnoses in the clinic (observed data) as red boxes.

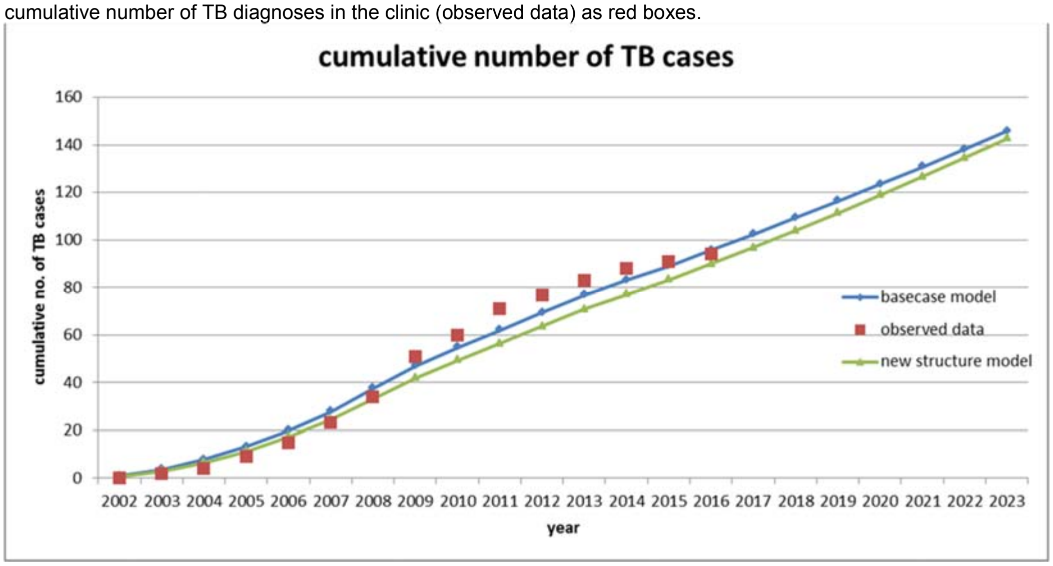

© 2019 Wong NS et al. *JAMA Network Open.*

eFigure 9. Incremental Cost-Effectiveness Ratio (ICER Plane) of LTBI Screening Strategies Under Different Coverage of Antiretroviral Therapy, LTBI Testing and Treatment, and TB treatment, 2017-2023

Screening strategies include strategy A: baseline screening ONLY; strategy B: baseline screening AND annual testing for risk groups; strategy C: baseline screening AND biennial testing for all until tested LTBI positive; strategy D: baseline screening AND at most three subsequent LTBI tests; and strategy E: baseline screening AND annual testing for all until tested LTBI positive. These strategies are under different coverage of antiretroviral therapy (ART), LTBI testing and treatment and TB treatment, including Scenario 1: baseline value for all; scenario 2: 100% coverage for all; scenario 3: baseline value for LTBI testing, and 100% for the rest; scenario 4: baseline value for LTBI testing and ART, and 100% for LTBI treatment and TB treatment; and scenario 5: baseline value for ART, and 100% for the rest. The incremental cost and quality-adjusted life-years (QALY) gained were compared with status quo (scenario E1).

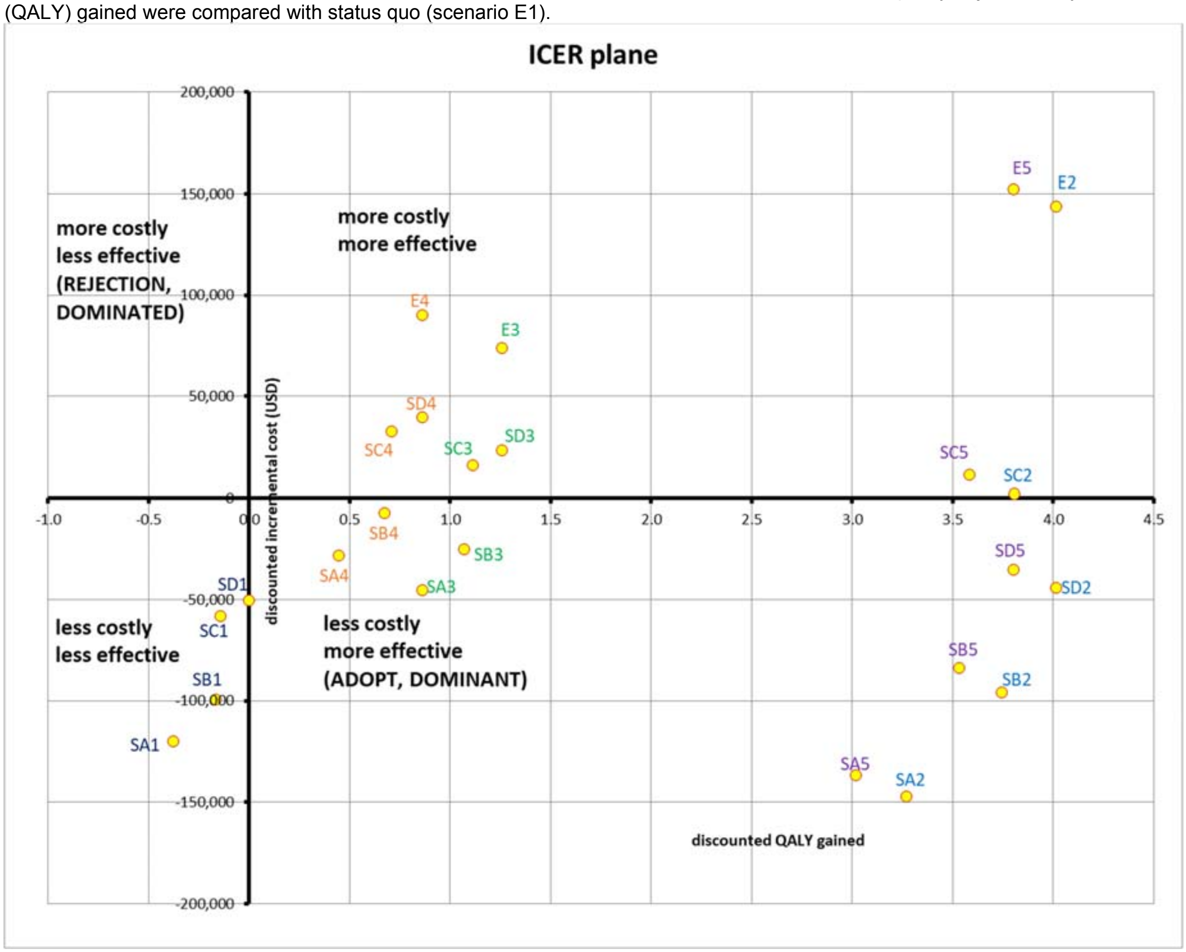

© 2019 Wong NS et al. *JAMA Network Open.*

eFigure 10. Impact of Annual Number of Newly Diagnosed HIV Cases in the Clinic, LTBI Prevalence Among HIV-Positive Patients, and Reduction of TB Reactivation by LTBI Treatment on Incremental Cost-Effectiveness Ratio Under the Current Annual Testing Strategy at Baseline Coverage Value (Scenario E1) in 2017-2023

Results are shown for scenario E1 (annual testing for all, baseline coverage of ART, LTBI testing, LTBI treatment and TB treatment), with the relative change of (a) to (c) parameter's value from 0% to -90%, with -10% interval. The cost and QALY gained is compared with results in scenario E1.

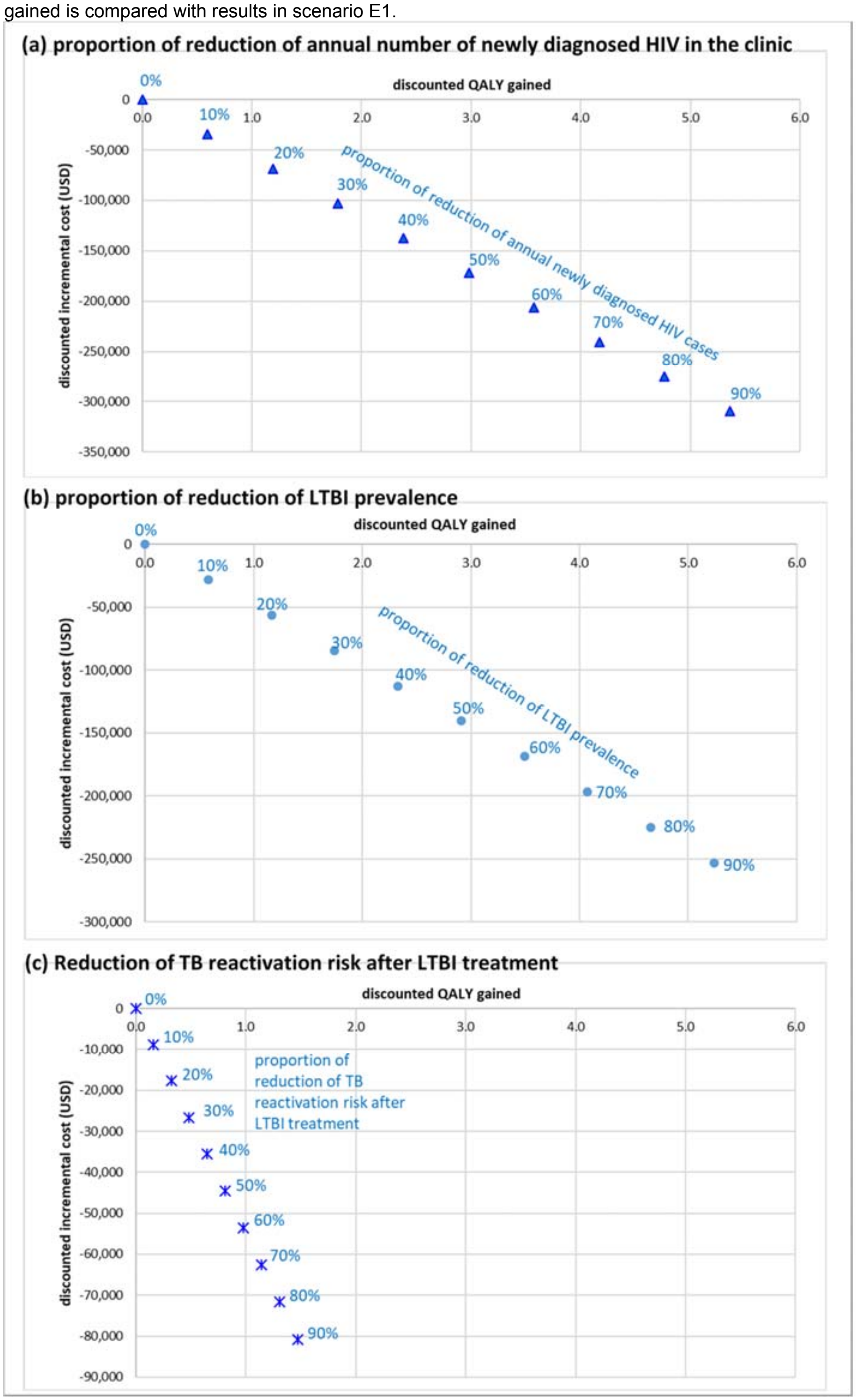

QALY - quality-adjusted life-years

© 2019 Wong NS et al. *JAMA Network Open.*

eFigure 11. Impact of LTBI Testing Coverage (40%-100%) and LTBI Treatment Coverage (50%-100%) Under Current Annual Testing Strategy at Baseline Coverage Value (Scenario E1) on Incremental Cost-Effectiveness Ratio (ICER) in 2017-2023, 2-way Sensitivity Analysis Results

Results are shown for scenario E1 (annual testing or all, baseline coverage of ART, LTBI testing, LTBI treatment and TB treatment), except change of LTBI testing and treatment coverage parameters. The cost and QALY gained is compared with status quo (results in scenario E1). The lower panel includes the ICER of other scenarios.

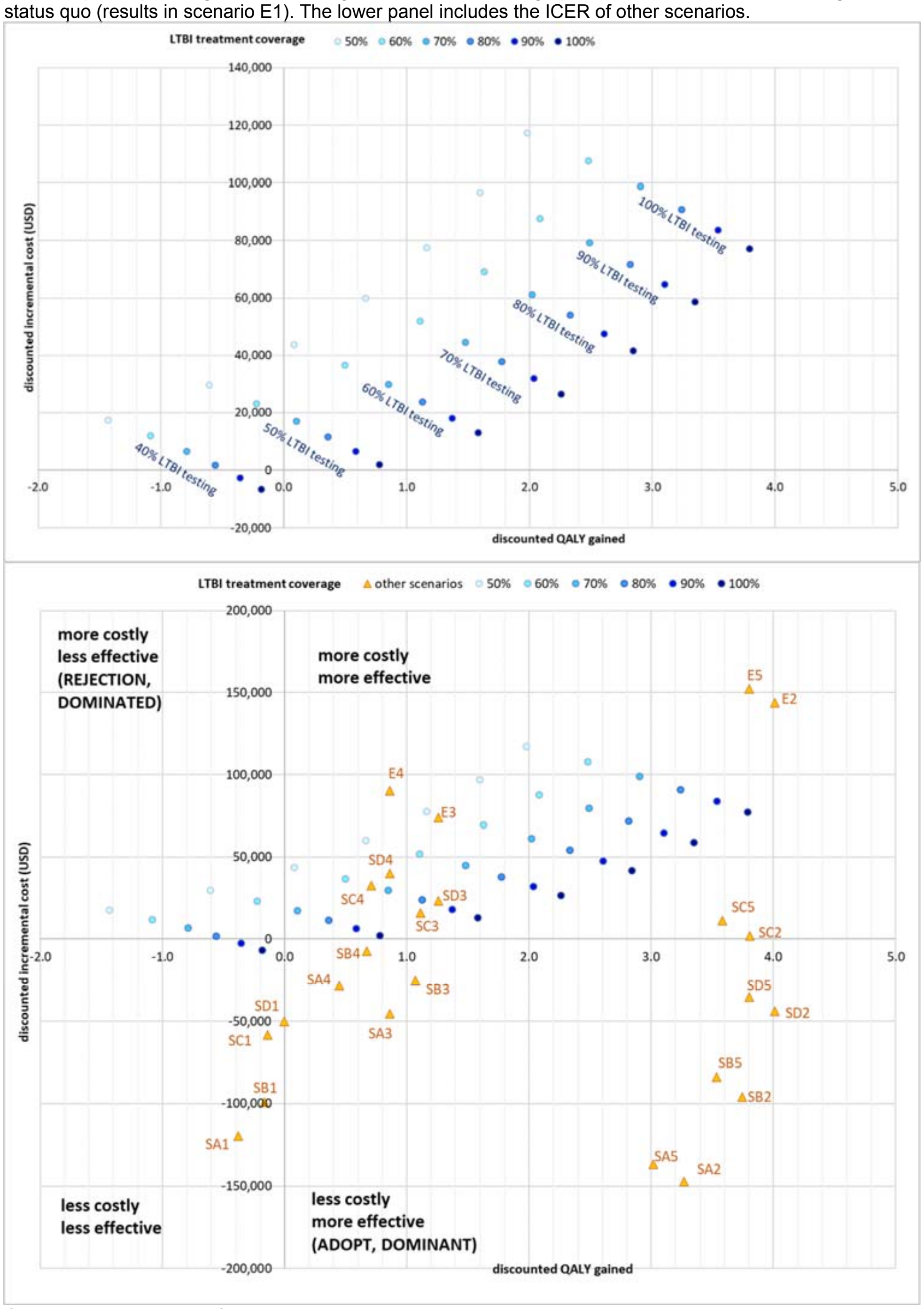

QALY - quality-adjusted life-years

© 2019 Wong NS et al. *JAMA Network Open.*

eFigure 12. Impact of LTBI Testing Coverage (40%-100%) and LTBI Treatment Coverage (50%-100%) Under at Most 3 Subsequent LTBI Testing Strategy (Scenario D) with 100% Antiretroviral Therapy Coverage on Incremental Cost-Effectiveness Ratio (ICER) in 2017-2023, 2-way Sensitivity Analysis Results

Results are shown for Scenario D2 (at most three subsequent LTBI testing strategy, 100% antiretroviral therapy, LTBI testing and treatment and TB treatment coverage), except change of LTBI testing and treatment coverage parameters. The cost and QALY gained is compared with status quo (results in scenario E1). The lower panel includes the ICER of other scenarios.

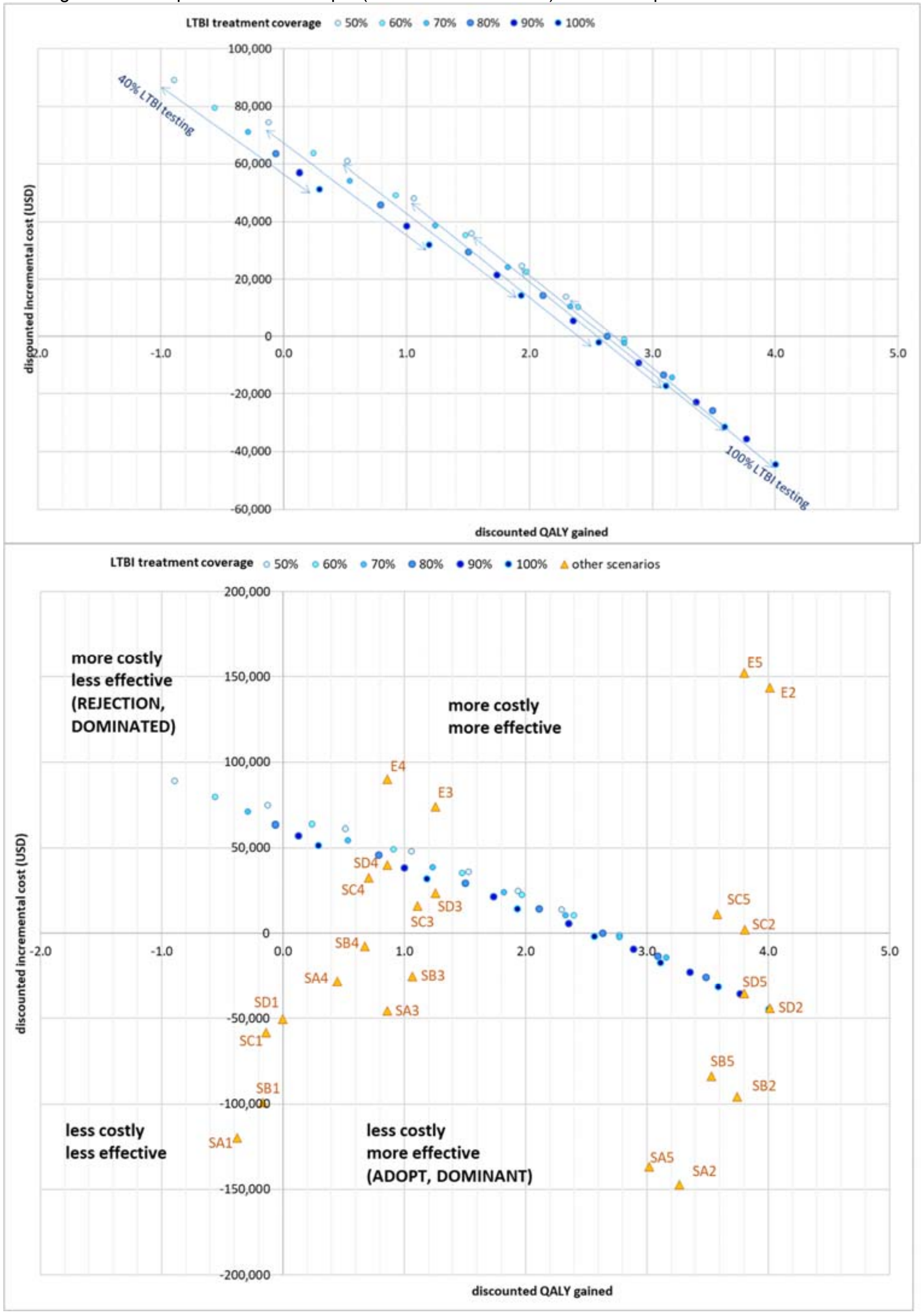

QALY - quality-adjusted life-years

© 2019 Wong NS et al. *JAMA Network Open.*

eFigure 13. Impact of LTBI Testing Coverage (40%-100%) and LTBI Treatment Coverage (50%-100%) Under Strategy of Testing by Risk Factor (Scenario B) With 100% Antiretroviral Therapy Coverage on Incremental Cost-Effectiveness Ratio (ICER) in 2017-2023, 2-way Sensitivity Analysis Results

Results are shown for Scenario B2 (testing by risk factors, 100% antiretroviral therapy, LTBI testing and treatment and TB treatment coverage), except change of LTBI testing and treatment coverage parameters. The cost and QALY gained is compared with status quo (results in scenario E1). The lower panel includes the ICER of other scenarios.

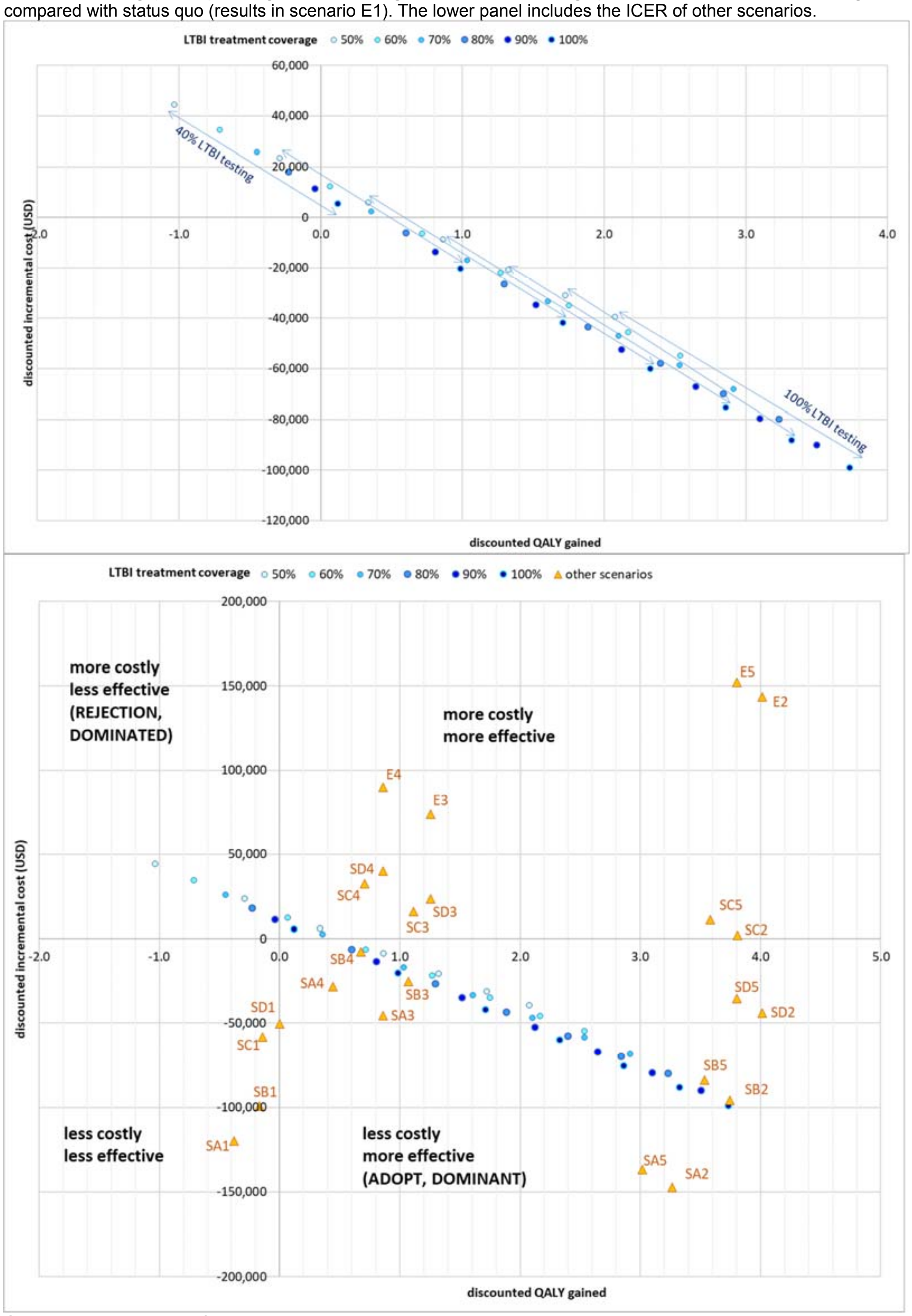

QALY - quality-adjusted life-year

© 2019 Wong NS et al. *JAMA Network Open.*

eReferences

[1] World Health Organization. *Latent tuberculosis infection: updated and consolidated guidelines for programmatic management*. 2018.

[2]. Lin AW, Lau SK, Woo PC. Screening and treatment of latent tuberculosis infection among HIV-infected patients in resource-rich settings. *Expert Rev Anti Infect Ther*. 2016;14(5):489-500.

[3] Tuberculosis & Chest Service / Special Preventive Programme, Hong Kong SAR Government. *Surveillance Report on TB/HIV co-infection in Hong Kong 2015*. http://www.info.gov.hk/tb_chest/doc/TB-HIV_Registry_2015.pdf

[4] Tuberculosis and Chest Service, Department of Health, the Government of Hong Kong Special Administrative Region. *Tuberculosis Manual*. 2006.

[5] Lin AW, Chan KC, Chan WK, Wong KH. Tuberculin sensitivity testing and treatment of latent tuberculosis remains effective for tuberculosis control in human immunodeficiency virus-infected patients in Hong Kong. *Hong Kong Med J*. 2013;19(5):386-92.

[6] Wong NS, Kwan TH, Tsang OTY, et al. Pre-exposure prophylaxis (PrEP) for MSM in low HIV incidence places: should high risk individuals be targeted? *Scientific Reports*. 2018:8(1):11641.

[7] Diel R, Hittel N, Schaberg T. Cost effectiveness of treating multi-drug resistant tuberculosis by adding Deltyba™ to background regimens in Germany. *Respir Med*. 2015;109(5):632-41.

© 2019 Wong NS et al. *JAMA Network Open.*
